# Integrated Pharmacogenomic and Structure-Guided Analyses Link LCC-10 (NSC765599) to an MMP-Associated Extracellular Matrix Regulatory Network in Leukemia

**DOI:** 10.3390/cells15171610

**Published:** 2026-09-04

**Authors:** Han-Lin Hsu, Tawakalitu Bidemi Aliu, Ya-Ting Wen, Yu-Cheng Kuo, Li Wei, Ruey-Shyang Soong, Maryam Rachmawati Sumitra, Sheng-Liang Huang, Shih-Yu Lee, Sung-Ling Tang, I-Chuan Yen, Hong-Jaan Wang, Bashir Lawal, George Hsiao, Alexander T. H. Wu, Hsu-Shan Huang

**Affiliations:** 1 Graduate Institute of Medical Sciences, College of Medicine, Taipei Medical University, Taipei 11031, Taiwan; 94401@w.tmu.edu.tw; 2 Department of Internal Medicine, Wan Fang Hospital, Taipei Medical University, Taipei 11681, Taiwan; 3 Graduate Institute of Cancer Biology and Drug Discovery, College of Medical Science and Technology, Taipei Medical University, Taipei 11031, Taiwan; d621112007@tmu.edu.tw (T.B.A.); maryamrachma60@gmail.com (M.R.S.); 4 Division of Neurosurgery, Department of Surgery, Wan Fang Hospital, Taipei Medical University, Taipei 10608, Taiwan; 98142@w.tmu.edu.tw (Y.-T.W.); 109058@w.tmu.edu.tw (L.W.); 5 Division of Neurosurgery, Department of Surgery, College of Medicine, Taipei Medical University, Taipei 11031, Taiwan; 6 Department of Pharmacology, College of Medicine, Taipei Medical University, Taipei 11031, Taiwan; yuchengkuo@tmu.edu.tw (Y.-C.K.); geohsiao@tmu.edu.tw (G.H.); 7 Graduate Institute of Injury Prevention and Control, College of Public Health, Taipei Medical University, Taipei 11031, Taiwan; 8 Division of General Surgery, Department of Surgery, Shuang Ho Hospital, Taipei Medical University, New Taipei City 23561, Taiwan; kodlp62@gmail.com; 9 Department of Surgery, School of Medicine, College of Medicine, Taipei Medical University, Taipei 11031, Taiwan; 10 Graduate Institute of Aerospace and Undersea Medicine, National Defense Medical University, Taipei 11490, Taiwan; angelsapmt@gmail.com (S.-L.H.); leeshihyuno1@mail.ndmctsgh.edu.tw (S.-Y.L.); 11 School of Pharmacy, National Defense Medical University, Taipei 11490, Taiwan; tangling@mail.ndmutsgh.edu.tw (S.-L.T.); yenichuan@mail.ndmctsgh.edu.tw (I.-C.Y.); hongjaan@mail.ndmctsgh.edu.tw (H.-J.W.); 12 UPMC Hillman Cancer Center, University of Pittsburgh, Pittsburgh, PA 15260, USA; bashirlawal12@gmail.com; 13 The Ph.D. Program of Translational Medicine, College of Medical Science and Technology, Taipei Medical University, Taipei 11031, Taiwan; 14 Taipei Heart Institute (THI), Taipei Medical University, Taipei 11031, Taiwan; 15 Ph.D. Program in Biotechnology Research and Development, College of Pharmacy, Taipei Medical University, Taipei 11031, Taiwan

**Keywords:** leukemia, extracellular matrix, matrix metalloproteinases, bone marrow microenvironment, pharmacogenomics, systems pharmacology

## Abstract

**Highlights:**

**What are the main findings?**

- Integrated pharmacogenomic analyses converged on an MMP-associated extracellular matrix regulatory network linked to LCC-10 activity in leukemia.
- Structure-guided analyses suggest structural compatibility with representative MMP catalytic domains, while zebrafish assays support preliminary developmental tolerability.

**What are the implications of the main findings?**

- This study provides an integrated computational framework for generating testable mechanistic hypotheses from phenotypic drug-response data.
- LCC-10 represents a hypothesis-generating lead for future biochemical, target-engagement, and functional validation in leukemia.

**Abstract:**

Leukemia progression is increasingly shaped by reciprocal interactions between leukemic cells and the bone marrow microenvironment, yet the extracellular regulatory networks associated with these interactions remain incompletely understood. Here, we investigated the biological context associated with the antileukemic activity of LCC-10 (NSC765599), a synthetic biphenyl benzamide derivative, using an integrated pharmacogenomic and structure-guided computational framework. Antiproliferative activity was first characterized using the NCI-60 screen and subsequently integrated with pharmacogenomic response similarity analysis, baseline transcriptomic profiling, similarity-based target prediction, systems-level network analysis, molecular docking, coarse-grained molecular dynamics simulations, comparative in silico ADMET evaluation, and zebrafish embryo developmental toxicity assessment. LCC-10 exhibited potent antiproliferative activity across leukemia cell lines, with submicromolar GI_50_ values in five of six models. Computational analyses converged on a matrix metalloproteinase (MMP)-associated extracellular matrix (ECM) regulatory network, with MMP2 and MMP9 among the recurrently implicated candidates. Structure-guided analyses suggested structural compatibility of LCC-10 with representative MMP catalytic domains but did not establish direct biochemical inhibition or target engagement. Comparative in silico ADMET analyses supported the predicted developability profile of LCC-10, whereas zebrafish embryo assays indicated concentration-dependent developmental tolerability within the tested range. Collectively, these findings associate LCC-10 with an MMP-associated ECM regulatory network in leukemia while defining this relationship as a hypothesis requiring direct experimental validation. This integrated framework provides a rationale for subsequent biochemical, target-engagement, and functional studies to clarify the molecular basis of LCC-10 activity.

## 1. Introduction

Leukemia comprises a heterogeneous group of hematological malignancies characterized by the uncontrolled proliferation of immature hematopoietic cells and remains a major cause of cancer-related mortality worldwide [1]. Despite substantial advances in chemotherapy, hematopoietic stem cell transplantation, molecularly targeted therapies, and, more recently, immunotherapy, long-term clinical outcomes remain limited by disease relapse, therapeutic resistance, and persistence of leukemia-initiating cells [2]. Increasing evidence indicates that leukemia progression is governed not only by intrinsic genetic and epigenetic alterations but also by dynamic interactions between leukemic cells and the bone marrow microenvironment [3]. In particular, the ECM provides structural support together with cytokines, growth factors, and proteolytic signals that collectively regulate leukemic cell survival, therapeutic tolerance, and disease progression [4]. These findings have increased interest in leukemia-supportive microenvironmental regulatory networks as potential complements to therapeutic strategies directed against leukemic cells themselves.

Natural products and their synthetic derivatives remain an important source of structurally diverse lead compounds for anticancer drug discovery [5]. Among these, honokiol, a biphenyl neolignan isolated from *Magnolia officinalis*, exhibits antiproliferative, anti-inflammatory, and pro-apoptotic activities across multiple malignancies, including leukemia [6,7,8]. Likewise, the FDA-approved salicylanilide niclosamide has attracted considerable attention because of its ability to modulate multiple oncogenic signaling pathways [9]. Fragment-inspired scaffold-hopping strategies have further enabled the development of chemically tractable small molecules with improved drug-like properties and expanded biological activities [10,11]. LCC-10 (NSC765599) is a synthetic biphenyl benzamide derivative developed in our laboratory through fragment-inspired scaffold-hopping optimization that combines a honokiol-derived biphenyl scaffold with a niclosamide-inspired benzamide pharmacophore. Although LCC-10 has previously been reported to inhibit receptor activator of nuclear factor-κB ligand (RANKL)-induced osteoclastogenesis [12], its antileukemic activity and the biological pathways associated with its pharmacological effects remain largely unknown.

Recent advances in systems pharmacology and computational biology have transformed phenotypic drug discovery by enabling biological hypotheses to be generated from multidimensional pharmacogenomic datasets without requiring prior knowledge of molecular targets [13]. The NCI-60 represents one of the best-characterized phenotypic-molecular platforms for integrating drug-response profiles with genomic and transcriptomic information to facilitate mechanism-oriented drug discovery [14]. Rather than relying on a single prediction algorithm, integration of complementary and partially interdependent computational approaches can identify biological pathways recurrently implicated across analytical layers, thereby providing a systems-level strategy for hypothesis generation [15]. Importantly, however, such computational convergence identifies candidate biological associations and does not by itself establish direct target engagement or causal mechanisms of drug action. Whether such an integrated framework can identify extracellular regulatory programs associated with the antileukemic activity of LCC-10 has not been investigated.

Here, we applied an integrated pharmacogenomic and structure-guided computational framework to investigate the biological programs associated with the antileukemic activity of LCC-10 (NSC765599). Antiproliferative activity was first characterized using the NCI-60 screen and subsequently integrated with pharmacogenomic response similarity analysis [16], baseline transcriptomic profiling [17], similarity-based target prediction [18], protein–protein interaction (PPI) network analysis [19], functional enrichment analysis [20], molecular docking [21], coarse-grained molecular dynamics simulations [22], comparativein silico ADMET evaluation [23], and zebrafish embryo developmental toxicity assessment [24]. Rather than beginning with a predefined molecular target, this response-driven strategy prioritized an MMP-associated ECM regulatory network through the sequential integration of complementary analytical layers [25]. We therefore evaluated representative MMP catalytic domains as one structurally testable component of this network, while recognizing that other predicted intracellular targets may also contribute to the observed antiproliferative phenotype. Collectively, our findings associate LCC-10 with an MMP-associated ECM regulatory network in leukemia and establish a hypothesis-generating framework for subsequent biochemical, target-engagement, and functional validation.

## 2. Materials and Methods

### 2.1. Compounds and Reagents

LCC-10 (NSC765599) was designed and synthesized in our laboratory as previously described [12]. Briefly, the synthesis involved a two-stage route starting from diflunisal. Diflunisal (Angene international limited, China) was first activated with thionyl chloride (Sigma-aldrich, USA) to generate the corresponding acid chloride, followed by coupling with the appropriate substituted aniline to afford the biphenyl benzamide intermediate. After purification by extraction and crystallization, the intermediate was further reacted with methyl chloroformate (Sigma-aldrich, USA) in tetrahydrofuran/pyridine (Thermo Scientific, Belgium) under controlled temperature, followed by reflux and mild acidic work-up, as described previously [12]. The resulting product was purified by crystallization from hot ethanol. Compound identity was confirmed by HRMS and ^1^H and ^13^C NMR spectroscopy, and chemical purity (>98%) was verified by HPLC. LCC-10 was subsequently submitted to the Developmental Therapeutics Program (DTP) of the U.S. National Cancer Institute (NCI), assigned the identifier NSC765599, and evaluated in the NCI-60 screen.

### 2.2. NCI-60 Drug-Response Profiling

The antiproliferative activity of NSC765599 was evaluated using publicly available data from the NCI-60 screen generated by the DTP of the U.S. NCI [26]. Cell growth was assessed using the SRB assay according to established NCI-DTP protocols (https://dtp.cancer.gov/discovery_development/nci-60/default.htm) accessed on May 2026. NSC765599 was initially screened at 10 μM and subsequently evaluated in a five-dose concentration–response assay. Pharmacological response parameters, including the GI_50_, TGI, LC_50_, and IC_50_, were retrieved directly from the NCI-DTP database [27]. Downstream analyses focused on the leukemia-associated cell lines CCRF-CEM, HL-60(TB), K-562, MOLT-4, RPMI-8226, and SR, incorporating both single-dose growth inhibition data and five-dose pharmacological response parameters.

### 2.3. Pharmacological Response Similarity Analysis Using DTP-COMPARE

Pharmacological response similarity analysis was performed using the DTP-COMPARE algorithm (https://dtp.cancer.gov/private_compare/) accessed on May 2026 to identify compounds with NCI-60 phenotypic response signatures similar to that of NSC765599 [28]. The single-dose (10 μM) growth inhibition profile of NSC765599 was used as the query signature. Compounds were ranked according to their Pearson correlation coefficients, and the highest-ranked compounds were retained for comparative analysis. The number of common cell lines contributing to each correlation was also recorded to assess the comparability of the underlying response patterns. The resulting similarity rankings were incorporated into the subsequent integrative workflow. Downstream transcriptomic profiling, similarity-based target prediction, PPI network construction, functional enrichment, and structure-guided analyses were used as complementary, although not necessarily independent, analytical layers for hypothesis generation.

### 2.4. Baseline Transcriptomic Profiling of Leukemia-Associated Cell Lines

Baseline transcriptomic data were obtained from the CellMiner platform (https://discover.nci.nih.gov/cellminer/) accessed on May 2026 [29,30]. Relative mRNA expression levels of representative MMP family members, including MMP2, MMP3, MMP9, MMP12, and MMP13, were retrieved for the leukemia cell lines CCRF-CEM, HL-60(TB), K-562, MOLT-4, RPMI-8226, and SR. Expression data were visualized to compare baseline MMP expression patterns across the leukemia cell lines [31]. These analyses were descriptive and were used solely to characterize the baseline expression of candidate MMPs; they were not interpreted as evidence of an association between MMP expression and LCC-10 sensitivity, LCC-10-induced transcriptional modulation, or direct target engagement.

### 2.5. Similarity-Based Target Prediction Using SwissTargetPrediction

Similarity-based target prediction was performed using the SwissTargetPrediction web server (https://www.swisstargetprediction.ch/) accessed on May 2026 to identify candidate protein targets of LCC-10 [32]. SwissTargetPrediction estimates potential targets based on 2D and 3D molecular similarity between the query compound and ligands with experimentally characterized targets [33]. The canonical SMILES representation of LCC-10 was submitted using Homo sapiens as the reference species with default parameters. Predicted targets were ranked according to their prediction probabilities and classified by protein class. The complete predicted target landscape, including both proteases and alternative intracellular signaling proteins, was retained for comparative interpretation (Supplementary Table S2). Representative MMP family members were subsequently selected for structure-guided evaluation because they provided a structurally testable component of the ECM-associated hypothesis emerging from the sequentially integrated analyses. Alternative predicted targets, particularly intracellular signaling proteins, were retained as plausible contributors to the pharmacological activity of LCC-10 and were not excluded by the present analysis [34].

### 2.6. PPI Network and Functional Enrichment Analyses

PPI and functional enrichment analyses were performed to characterize the systems-level biological context associated with the candidate targets of LCC-10. Candidate proteins identified through similarity-based target prediction, together with representative MMP family members examined in the ECM-associated analyses, were subjected to STRING-based PPI network analysis using STRING version 12.0, with Homo sapiens selected as the reference organism [35]. Protein associations were evaluated using a minimum interaction confidence score of 0.70 [36]. Functional enrichment analyses were performed using Gene Ontology (GO) annotations, including biological process (BP), cellular component (CC), and molecular function (MF), together with Kyoto Encyclopedia of Genes and Genomes (KEGG) pathway analysis to characterize significantly enriched biological functions and signaling pathways [37]. Statistical significance was assessed using false discovery rate (FDR)-adjusted *p*-values, with FDR < 0.05 considered significant [38]. Cancer Hallmarks Analytics Tool (CHAT) analysis was additionally performed to examine associations between the MMP-associated network and established cancer hallmark processes, including tumor-promoting inflammation, angiogenesis, invasion and metastasis, proliferative signaling, and immune-related processes [39]. These network and enrichment analyses were used to characterize systems-level biological associations and to inform the selection of representative proteins for subsequent structure-guided evaluation; they were not interpreted as evidence of direct target engagement or pathway modulation by LCC-10.

### 2.7. Structure-Based Molecular Docking and Conformational Flexibility Analyses

Structure-based molecular docking analyses were performed to evaluate the predicted structural compatibility between LCC-10 and representative MMP catalytic domains. Five representative MMP family members (MMP2, MMP3, MMP9, MMP12, and MMP13), together with four reference MMP inhibitors (batimastat, marimastat, PD166793, and SB-3CT) were included for comparative analyses [40]. Three-dimensional crystal structures were retrieved from the Protein Data Bank (PDB) and prepared by removing crystallographic water molecules, adding hydrogen atoms, and assigning atomic charges where appropriate [41]. Molecular docking simulations were performed using AutoDock Vina v1.2.5 [42]. Docking grid boxes were centered on the catalytic zinc-binding region or the co-crystallized ligand-binding pocket of each MMP structure [43]. Predicted binding poses were ranked according to the Vina docking score (kcal/mol), and the top-ranked conformations were selected for subsequent interaction analysis. Protein–ligand interactions, including hydrogen bonds, hydrophobic contacts, π-interactions, halogen interactions, van der Waals contacts, and residues surrounding the catalytic pocket, were analyzed and visualized using BIOVIA Discovery Studio Visualizer v21.1.0.20298 and PyMOLv3.1.5.1 [44].

To complement the docking analysis, coarse-grained protein conformational flexibility analysis was performed using the CABS-flex platform [45]. Representative MMP protein structures corresponding to the structural models selected from the docking analyses of LCC-10 and the reference inhibitors were used as inputs. CABS-flex analyses were performed using the default simulation settings, including 100% retained restraints, 50 simulation cycles, 50 cycles between trajectory frames, and a simulation temperature of 1.4. A single CABS-flex analysis was performed for each structural model, and no replicate simulations were used for statistical inference. Because CABS-flex evaluates coarse-grained protein conformational flexibility rather than explicit protein–ligand molecular dynamics, the ligands were not treated as dynamically retained components within the binding pocket during the flexibility simulations. Residue-level root mean square fluctuation (RMSF) profiles were generated from the resulting conformational ensembles and used to compare local protein flexibility across the corresponding MMP structural models. Representative backbone conformational ensembles were also examined to identify regions of relatively greater or lower structural mobility. Because CABS-flex does not quantify ligand-binding free energies, residence times, dissociation kinetics, or ligand retention, these analyses were interpreted solely as computational assessments of protein conformational flexibility and structural compatibility rather than as direct evidence of ligand-binding stability, target engagement, or biochemical inhibition [46,47].

### 2.8. Physicochemical and In Silico ADMET Analyses

The physicochemical properties, drug-likeness, pharmacokinetic, and toxicity-related parameters of LCC-10 were predicted using SwissADME, ADMETLab 3.0, pkCSM, and GUSAR [48]. The canonical SMILES representation of LCC-10 was submitted to each platform using the default prediction settings. Predicted physicochemical descriptors included molecular weight, lipophilicity (LogP), topological polar surface area (TPSA), hydrogen-bond donor and acceptor counts, rotatable bonds, aqueous solubility, and other parameters relevant to oral drug-likeness [49]. Pharmacokinetic predictions included gastrointestinal absorption, blood–brain barrier permeability, P-glycoprotein substrate liability, and potential interactions with major cytochrome P450 isoforms [50]. Drug-likeness was evaluated according to Lipinski’s rule of five, Veber’s rule, predicted bioavailability score, and synthetic accessibility [51]. Predicted toxicity endpoints included Ames mutagenicity, human ether-à-go-go-related gene (hERG) liability, hepatotoxicity, acute toxicity, and additional platform-specific safety descriptors [52]. The same prediction workflow and parameter settings were applied to the reference MMP inhibitors (batimastat, marimastat, PD166793, and SB-3CT) for comparative analyses. The complete comparative ADMET prediction dataset is provided in Supplementary Table S4. These in silico analyses were used to comparatively assess physicochemical and predicted developability profiles and were not interpreted as evidence of clinical pharmacokinetics or safety in humans.

### 2.9. Zebrafish Embryo Developmental Toxicity Assay

Zebrafish embryo developmental toxicity assays were performed to evaluate the preliminary developmental tolerability of LCC-10. Wild-type AB zebrafish were maintained at the Core Laboratory of Zebrafish, Taipei Medical University (TMU), Taipei City, Taiwan, under standard husbandry conditions in accordance with institutional animal care guidelines [24]. Fertilized embryos obtained by natural spawning were maintained in E3 embryo medium. At 6 h post-fertilization (hpf), embryos were transferred to 24-well plates (10 embryos per well; three replicate wells per treatment group; 30 embryos per treatment group) and exposed to vehicle control or LCC-10 at final concentrations of 3, 6, or 9 μM. Exposure media were renewed daily throughout the experimental period. Embryos were examined at 24, 48, 72, and 96 hpf for survival, hatching rate, gross morphology, and developmental progression. Developmental endpoints, including body length, eye area, yolk sac area, and heart rate, were quantified at 96 hpf. Bright-field images were acquired using an Olympus SZX16 stereomicroscope, and quantitative image analysis was performed using ImageJ v1.54d-assisted image analysis (National Institutes of Health, Bethesda, MD, USA). This assay was used exclusively as a developmental toxicity/tolerability screen and was not designed to evaluate antileukemic efficacy or mechanism of action. This study was approved by the TMU Institutional Animal Care and Use Committee (Certificate No. 2025_45).

### 2.10. Statistical Analysis

Statistical analyses were performed using GraphPad Prism (version 10.4.2; GraphPad Software, San Diego, CA, USA). Pharmacological response similarity was assessed using Pearson correlation coefficients generated by the DTP-COMPARE algorithm from the single-dose (10 μM) NCI-60 growth-inhibition profiles. Log-transformed GI_50_ values retrieved from the NCI-DTP database were used for comparative analyses across the leukemia-associated cell lines. Functional enrichment significance was assessed using FDR-adjusted *p*-values, with FDR < 0.05 considered statistically significant. Zebrafish developmental toxicity data are presented as the mean ± standard deviation (SD). Each treatment group consisted of three replicate wells, with 10 embryos per well. For survival and hatching outcomes, the replicate well was treated as the independent experimental unit, with individual embryos within each well considered subsamples. For morphometric endpoints, measurements were obtained from individual embryos within each replicate well and summarized at the well level for statistical analysis, such that the replicate well, rather than the individual embryo, constituted the independent experimental unit. Statistical comparisons among treatment groups for morphometric endpoints were performed using one-way analysis of variance (ANOVA), followed by Tukey’s multiple-comparison test for comparisons of each LCC-10 treatment group with the vehicle control. All statistical tests were two-sided, and *p* < 0.05 was considered statistically significant.

## 3. Results

### 3.1. NSC765599 Exhibits Potent Antiproliferative Activity in Leukemia-Associated Cell Lines

**Figure 1:**
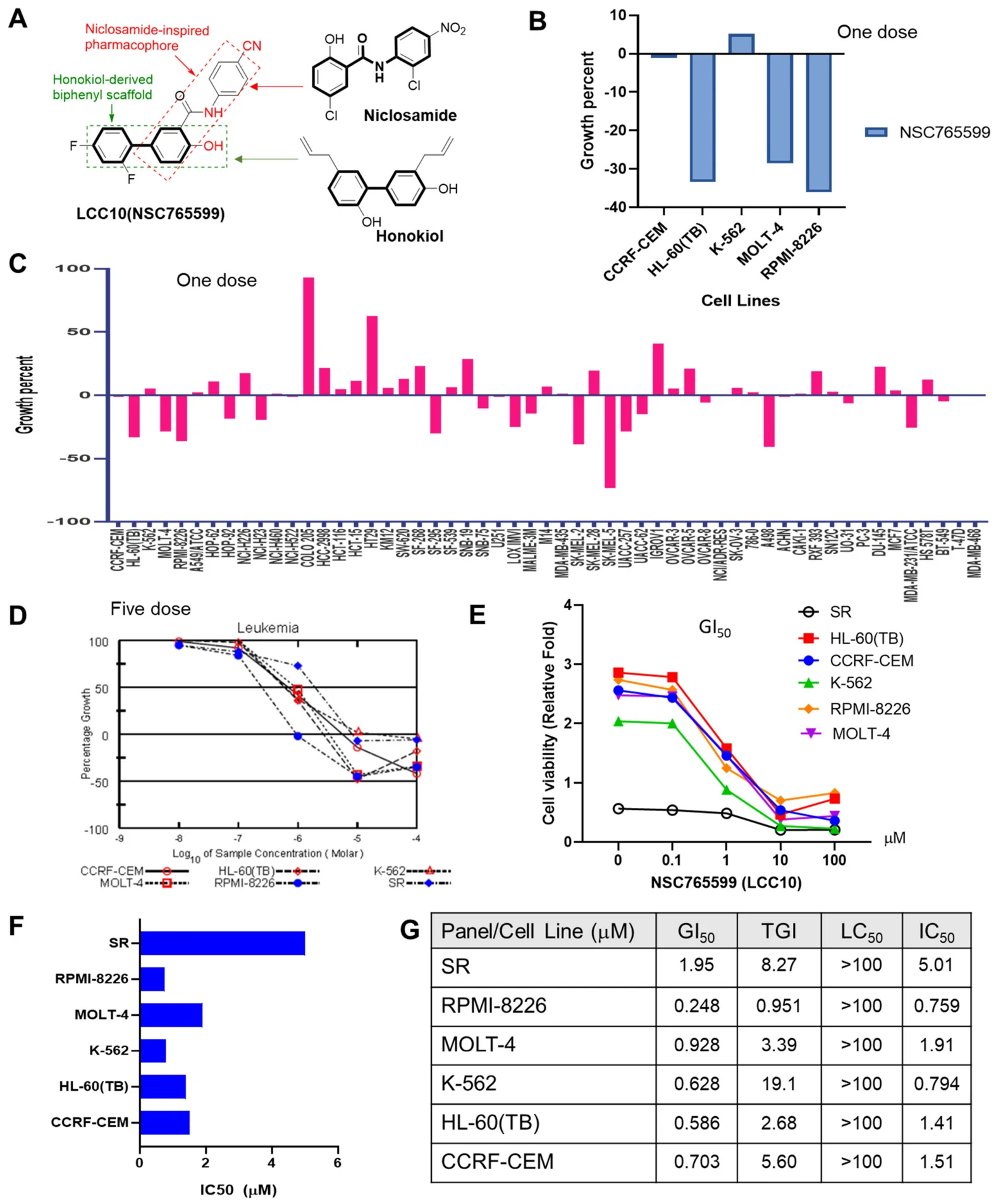
Antiproliferative activity of NSC765599 across the NCI-60 panel. (**A**) Chemical structures of NSC765599 illustrating its fragment-inspired design incorporating a honokiol-derived biphenyl scaffold and a niclosamide-inspired pharmacophore. (**B**) Single-dose (10 μM) growth-response profileof NSC765599 across the leukemia-associated NCI-60 cell lines. (**C**) Single-dose (10 μM) growth-response profile of NSC765599 across the complete NCI-60 panel. (**D**) Five-dose concentration–response profiles of NSC765599 across the six leukemia-associated cell lines. (**E**) Comparative GI_50_ values of NSC765599 across leukemia-associated cell lines. (**F**) Comparative IC_50_ values across the six leukemia-associated cell lines. (**G**) Summary of pharmacological response parameters (GI_50_, TGI, LC_50_, and IC_50_) for the six leukemia-associated cell lines.

NSC765599, a synthetic biphenyl benzamide incorporating structural features derived from honokiol and niclosamide, was evaluated using the NCI-60 panel to characterize its antiproliferative activity (Figure 1). Initial single-dose screening (10 μM) demonstrated broad growth-inhibitory activity across multiple cancer types, with the leukemia subpanel exhibiting marked sensitivity to NSC765599. Among the leukemia-associated cell lines, CCRF-CEM, HL-60(TB), K-562, MOLT-4, and RPMI-8226 showed substantial growth inhibition, with heterogeneous responses observed across the broader NCI-60 panel. Five-dose concentration–response analyses further demonstrated concentration-dependent growth inhibition across all six leukemia-associated cell lines, including CCRF-CEM, HL-60(TB), K-562, MOLT-4, RPMI-8226, and SR. GI_50_ values were submicromolar in five of the six leukemia-associated cell lines, ranging from 0.248 to 0.928 μM, whereas SR exhibited a GI_50_ value of 1.95 μM. Corresponding IC_50_ values ranged from 0.759 to 5.01 μM, with RPMI-8226, HL-60(TB), and MOLT-4 displaying the greatest sensitivity to NSC765599. TGI was achieved at low-to-moderate micromolar concentrations, whereas LC_50_ values generally exceeded 100 μM across the leukemia subpanel. Collectively, these findings define the phenotypic antiproliferative profile of NSC765599 in leukemia-associated cell lines and provide the basis for subsequent pharmacogenomic response-similarity, systems-level, and structure-guided computational analyses. Complete pharmacological response data for the NCI-60 panel are provided in Supplementary Figure S1.

### 3.2. DTP-COMPARE Analysis Identifies a Distinct Pharmacological Response Signature of NSC765599

To identify compounds exhibiting similar phenotypic response patterns, the NCI-60 growth-response profile of NSC765599 was analyzed using the DTP-COMPARE algorithm. Response-similarity analysis identified several investigational compounds with high pharmacological similarity (Pearson’s r = 0.76–0.92), most of which were structurally related biphenyl analogs. The clinically approved salicylanilide niclosamide also exhibited relatively high response similarity (r = 0.71), consistent with a partially shared NCI-60 phenotypic response pattern. The highest-ranked compounds displayed comparable common cell line counts (CCLC = 55–57), indicating that the correlations were derived from similarly represented NCI-60 datasets. In contrast, established cytotoxic and targeted anticancer agents, including platinum compounds, alkylating agents, topoisomerase inhibitors, antimetabolites, anthracyclines, and mTOR inhibitors, showed substantially lower response similarity (r = 0.12–0.29), despite comparable common cell line counts (Supplementary Table S1). Collectively, these findings indicate that NSC765599 displays an NCI-60 pharmacological response signature distinct from those of conventional anticancer agents examined. This phenotypic observation provided an initial pharmacological context that was subsequently integrated with baseline transcriptomic characterization, similarity-based target prediction, systems-level network analyses, and structure-guided computational evaluation, without assigning a specific molecular mechanism from the DTP-COMPARE profile alone.

### 3.3. Integrated Transcriptomic and Functional Enrichment Analyses Highlight an MMP-Associated ECM Regulatory Context

**Figure 2:**
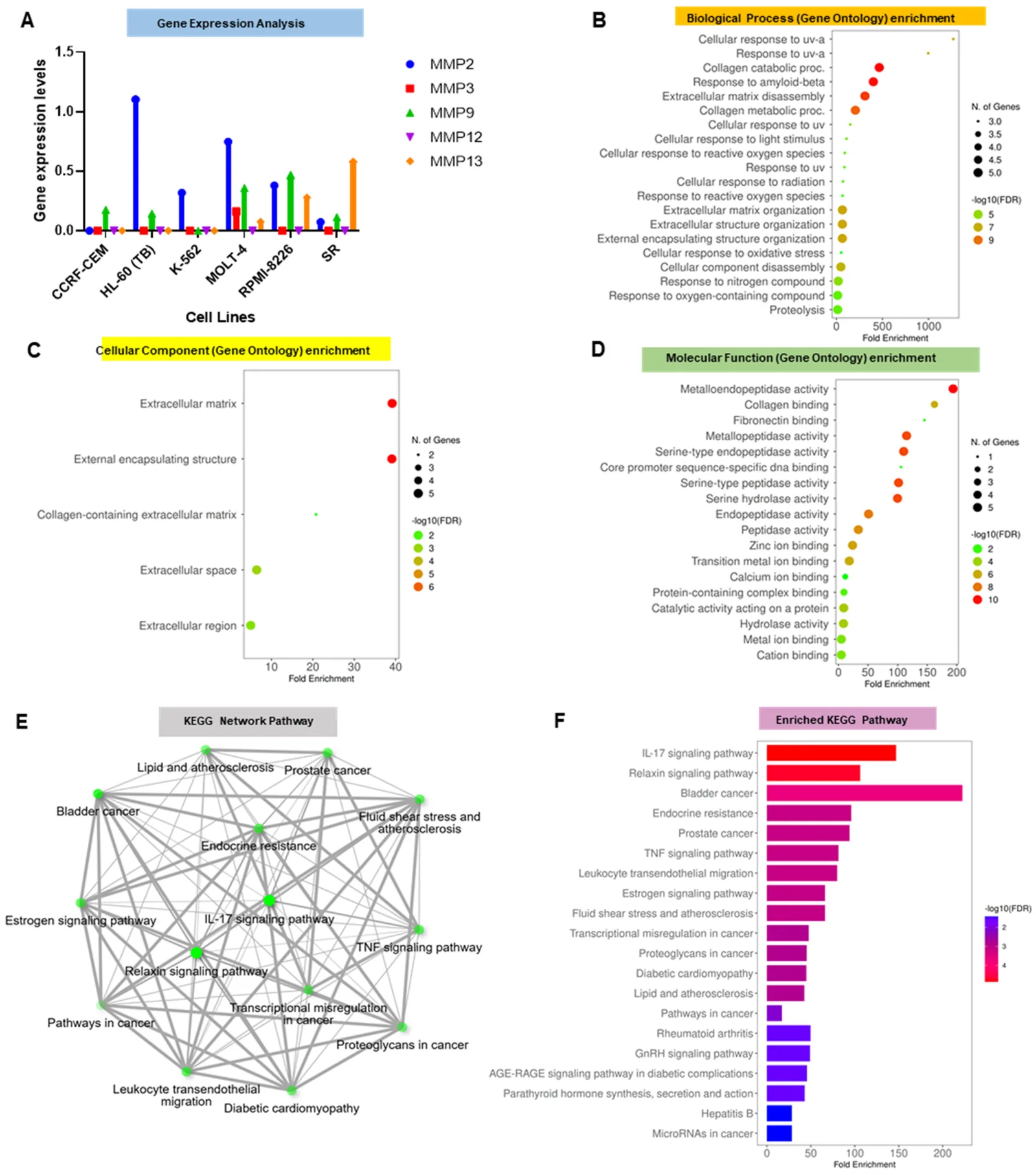
Integrated transcriptomic and functional enrichment analyses support an MMP-associated ECM regulatory context. (**A**) Baseline relative mRNA expression of MMP2, MMP3, MMP9, MMP12, and MMP13 across leukemia-associated NCI-60 cell lines retrieved from the CellMiner database. These data represent baseline expression in untreated cells and do not indicate LCC-10-induced transcriptional modulation. (**B**–**D**) Gene Ontology (GO) enrichment analyses showing significantly enriched biological process (BP), cellular component (CC), and molecular function (MF) categories, respectively. (**E**) KEGG pathway interaction network. (**F**) Ranked KEGG pathway enrichment analysis. Collectively, these analyses provide systems-level support for an MMP-associated ECM regulatory context within the candidate target network of LCC-10, without establishing direct target engagement or LCC-10-induced pathway modulation.

To characterize the biological context associated with the pharmacological activity of LCC-10, baseline transcriptomic expression profiles of representative MMPs (MMP2, MMP3, MMP9, MMP12, and MMP13) were examined across leukemia-associated cell lines using the CellMiner platform (Figure 2A) [29]. Individual MMP family members exhibited heterogeneous expression patterns across the leukemia models [53]. MMP2 and MMP9 were expressed across multiple cell lines, whereas MMP3, MMP12, and MMP13 displayed more cell line-specific expression patterns [54]. These CellMiner data represent baseline mRNA expression in untreated cells and therefore establish the presence and relative expression of candidate MMP-related genes across the leukemia models, rather than LCC-10-induced transcriptional modulation or direct target engagement [55,56]. Importantly, no direct expression–response relationship between individual MMPs and LCC-10 sensitivity is inferred from these data. Functional enrichment analyses provided complementary systems-level context for the candidate protein network [57]. GO biological process analysis identified significant enrichment of ECM-associated processes, including ECM organization, collagen catabolic process, extracellular structure disassembly, proteolysis, and cellular responses to oxidative and inflammatory stimuli (Figure 2B) [58]. Consistent with these findings, GO cellular component analysis identified the ECM, collagen-containing ECM, extracellular region, extracellular space, and external encapsulating structure among thepredominant enriched cellular compartments (Figure 2C). GO molecular function analysis further highlighted metalloendopeptidase activity, metallopeptidase activity, collagen binding, fibronectin binding, and zinc ion binding, supporting enrichment of extracellular proteolytic functions associated with ECM remodeling (Figure 2D). KEGG pathway analysis further revealed enrichment of pathways associated with inflammatory signaling, extracellular remodeling, and cellular migration (Figure 2E). Among the enriched pathways were IL-17 signaling, TNF signaling, leukocyte transendothelial migration, proteoglycans in cancer, transcriptional misregulation in cancer, and fluid shear stress and atherosclerosis (Figure 2F) [59]. Additional enriched pathways included endocrine resistance, estrogen signaling, and several cancer-associated pathways, reflecting the broader biological functions represented within the predicted target network [60]. Collectively, the baseline expression and functional enrichment analyses highlighted an MMP-associated ECM regulatory context as a systems-level biological association in the leukemia models. Importantly, these analyses identify biological associations rather than LCC-10-induced pathway modulation. Together with the pharmacological response analyses, these observations provided a rationale for including representative MMP family members in the subsequent target-prediction and structure-guided analyses, while recognizing that these analytical layers are complementary rather than fully independent.

### 3.4. Similarity-Based Target Prediction Identifies Multiple Candidate Target Classes for LCC-10

To identify candidate protein targets potentially associated with LCC-10, similarity-based target prediction was performed using SwissTargetPrediction [32]. The predicted target profile encompassed multiple functional classes, including proteases, receptor tyrosine kinases, serine/threonine kinases, other enzymes, and secreted proteins. Among the predicted protease targets, several MMP family members, including MMP2, MMP3, MMP9, MMP12, and MMP13, were identified [61]. Importantly, theprediction alsoidentified several cancer-relevant intracellular signaling proteins, including the receptor tyrosine kinases EGFR, FGFR1, PDGFRB, and KDR; the serine/threonine kinases MAPK14, MTOR, GSK3B, PDK1, CHEK1, and MAPK1; and the non-receptor kinases SRC and PTK2B; together with PTGES and VEGFA (Supplementary Table S2) [62]. Thus, similarity-based prediction did not support a unique molecular target for LCC-10 but instead suggested a potentially multi-target pharmacological profile encompassing both extracellular proteases and intracellular signaling proteins. Within this broader predicted target landscape, representative MMP family members were prioritized for subsequent structure-guided analyses because the protease class converged with the baseline MMP expression and ECM/proteolysis-related enrichment patterns described above. This prioritization was intended to examine one systems-level hypothesis rather than to exclude alternative candidate mechanisms. Predicted intracellular targets, including MTOR, CHEK1, and EGFR, therefore remain plausible contributors to the antiproliferative phenotype and require direct experimental validation. Accordingly, subsequent docking and conformational analyses evaluated the structural compatibility of LCC-10 with representative MMP catalytic domains rather than establishing MMPs as exclusive or experimentally validated molecular targets.

### 3.5. Systems-Level Network Analyses Support an MMP-Associated ECM Regulatory Network

**Figure 3:**
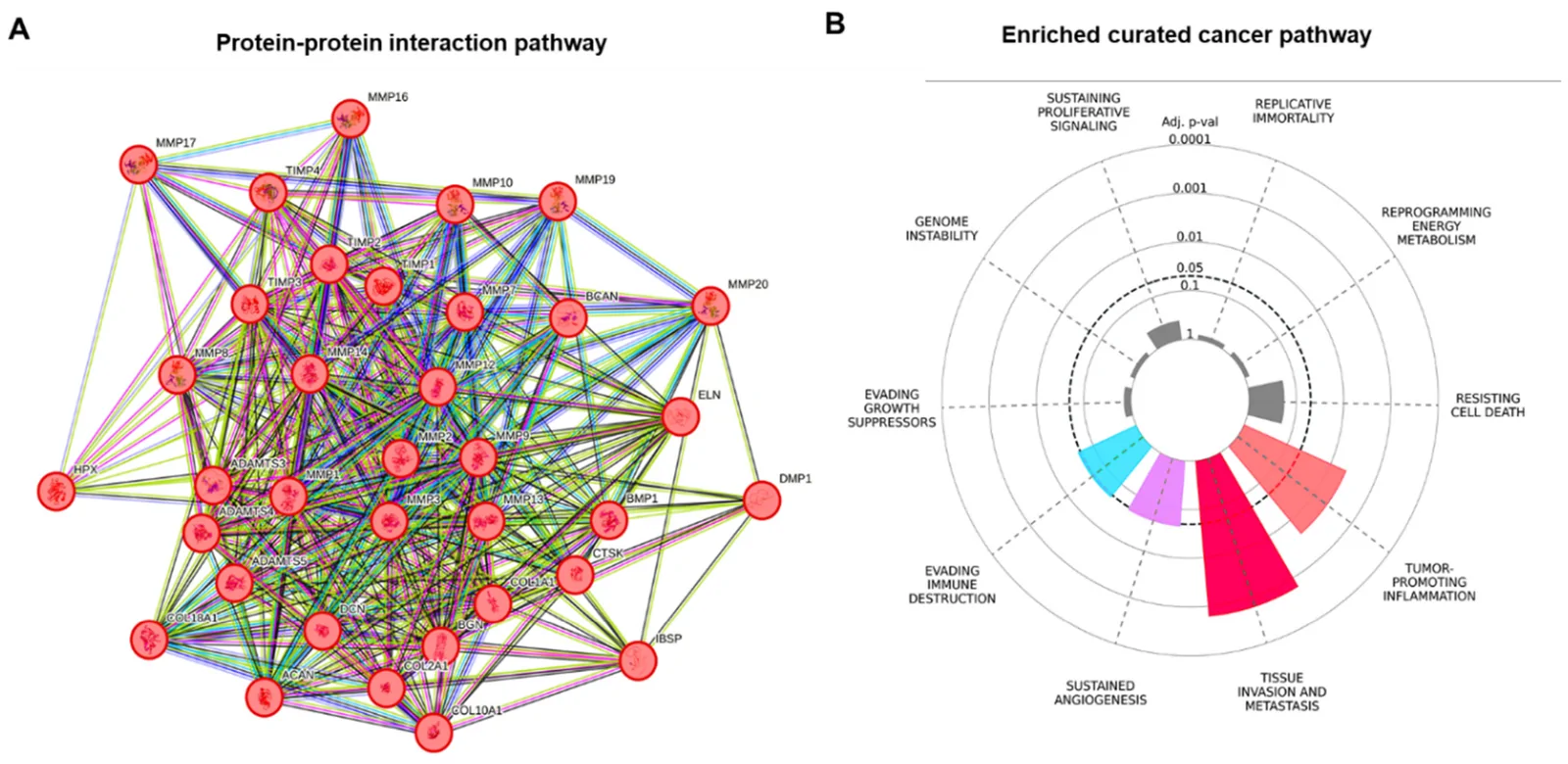
Systems-level network analyses support an MMP-associated ECM regulatory network. (**A**) High-confidence PPI network generated using the STRING database. Nodes represent proteins, and edges represent known or predicted functional associations among proteins. Representative MMP family members show extensive functional connectivity with ECM components, TIMPs, ADAM family metalloproteases, and other extracellular proteolytic regulators. (**B**) Cancer hallmark enrichment analysis of the MMP-associated network using curated hallmark gene sets. Radial bars indicate enrichment significance expressed as −log10 (adjusted *p*-values) across individual hallmark categories. Prominent enrichment was observed in tissue invasion and metastasis, tumor-promoting inflammation, sustained angiogenesis, resistance to cell death, immune-related processes, proliferative signaling, metabolic reprogramming, and genome instability. These network-level associations support an MMP-associated ECM regulatory context within the predicted target profile of LCC-10 but do not establish direct pathway modulation or target engagement. The different bar colors are used only to visually distinguish the individual cancer hallmark categories and do not represent an additional quantitative variable.

To further characterize the systems-level context of the candidate targets associated with of LCC-10, PPI network analysis was performed using the STRING database [63]. The resulting network demonstrated extensive functional connectivity among ECM remodeling proteins, MMPs, tissue inhibitors of metalloproteinases (TIMPs) [64], ADAM family metalloproteases [65], ECM components, and other protease-associated regulatory proteins (Figure 3A) [25]. Representative MMP family members, including MMP2, MMP3, MMP9, MMP12, and MMP13, occupied interconnected positions within this network and showed associations with TIMPs, ADAM family members, and ECM-related proteins, supporting a coordinated extracellular proteolytic and ECM-remodeling network rather than isolated involvement of individual MMPs. To further characterize the biological functions represented within this network, cancer hallmark enrichment analysis was performed using curated hallmark gene sets. Significant enrichment was observed in processes associated with tissue invasion and metastasis, tumor-promoting inflammation, sustained angiogenesis, resistance to cell death, and immune-related regulation (Figure 3B). Additional enrichment involved proliferative signaling, replicative immortality, metabolic reprogramming, and genome instability, indicating that the predicted network encompasses biological processes extending beyond extracellular proteolysis alone. Importantly, these network-level associations were consistent with the ECM-related functional enrichment patterns identified in Figure 2 and with the similarity-based target-prediction results (Supplementary Table S2). However, because these analyses are based on predicted targets, baseline expression profiles, and functional associations, they do not establish LCC-10-induced pathway modulation or direct engagement of individual MMPs. Rather, the recurrence of MMP-/ECM-related features across analytical layers provided the rationale for prioritizing representative MMP catalytic domains for subsequent structure-based analyses. Collectively, these findings support an MMP-associated ECM regulatory network as a candidate systems-level biological context associated with the pharmacological profile of LCC-10, while direct biochemical and functional validation will be required to determine whether this network contributes causally to its antileukemic activity.

### 3.6. Structure-Based Analyses Support Structural Compatibility of LCC-10 with Representative MMP Catalytic Domains

**Figure 4:**
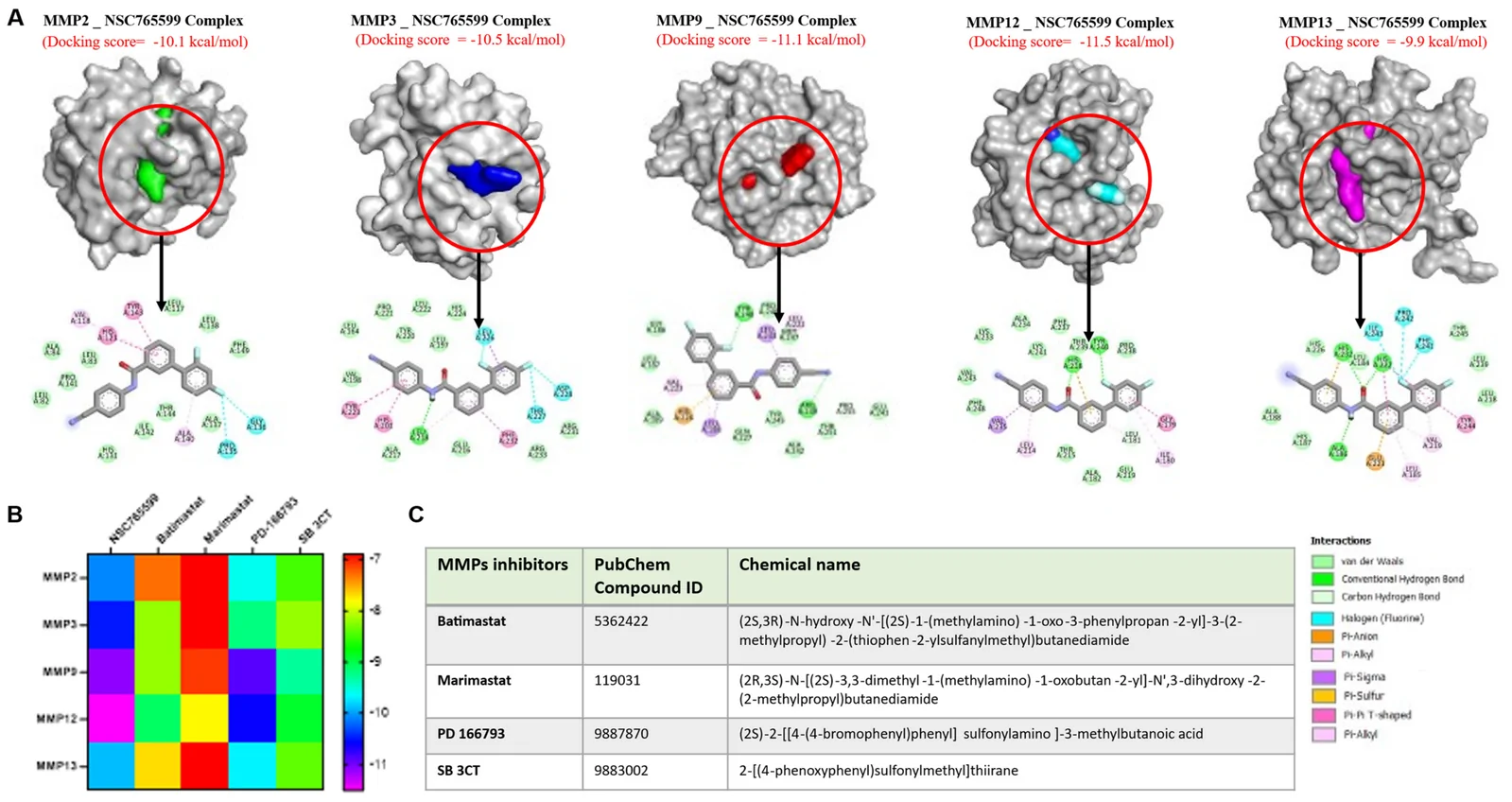
Structure-based molecular docking analyses support the structural compatibility of LCC-10 with representative MMP catalytic domains. (**A**) Surface representations of the predicted binding modes of LCC-10 within the catalytic domains of MMP2, MMP3, MMP9, MMP12, and MMP13. Predicted docking scores (kcal/mol) are indicated above each complex. LCC-10 was predicted to occupy the substrate-binding cavities and form multiple non-covalent contacts with residues surrounding the catalytic zinc-binding environment. Representative interacting residues and interaction types are summarized in Supplementary Table S3. (**B**) Heatmap comparing the predicted docking scores of LCC-10 and representative broad-spectrum MMP inhibitors (batimastat, marimastat, PD166793, and SB-3CT) across the five representative MMP isoforms. (**C**) Chemical structures, PubChem Compound IDs, and chemical names of the representative MMP inhibitors included in the comparative docking analyses.

To evaluate the structural compatibility of LCC-10 with representative MMP family members prioritized through the preceding pharmacogenomic, transcriptomic, and systems-level analyses, molecular docking was performed against five representative MMP catalytic domains (MMP2, MMP3, MMP9, MMP12, and MMP13) (Figure 4A). Representative broad-spectrum MMP inhibitors, including batimastat, marimastat, PD166793, and SB-3CT, were analyzed in parallel as structural comparators (Figure 4B,C) [66]. Across the five representative MMPs, LCC-10 yielded predicted binding energies ranging from −9.9 to −11.5 kcal/mol, with the most favorable docking scores observed for MMP12 (−11.5 kcal/mol) and MMP9 (−11.1 kcal/mol). The relatively narrow distribution of docking scores indicated broadly similar predicted structural compatibility across the five MMP catalytic domains rather than marked computational preference for a single MMP isoform. Inspection of the predicted docking poses suggested that LCC-10 could be accommodated within the catalytic pockets of all five analyzed MMPs, occupying the substrate-binding cavity and forming multiple predicted non-covalent contacts with residues surrounding the catalytic zinc-binding environment. These predicted interactions included hydrophobic contacts, conventional hydrogen bonds, halogen interactions, π-interactions, and van der Waals contacts, with representative interacting residues summarized in Supplementary Table S3. Comparative docking analyses indicated that the predicted docking scores of LCC-10 were within a range comparable to those obtained for the representative broad-spectrum MMP inhibitors and were numerically more favorable for several MMP isoforms (Figure 4B). Whereas the reference compounds showed greater variability in predicted docking scores across the five MMPs, LCC-10 displayed a relatively consistent computational docking profile. These comparisons are intended solely to provide structural reference points and should not be interpreted as evidence that LCC-10 has biochemical inhibitory potency comparable or superior to that of established MMP inhibitors. Collectively, the docking results complement the pharmacogenomic, transcriptomic, target prediction, and systems-level network analyses presented above by suggesting that LCC-10 is computationally compatible with the catalytic pockets of representative MMP family members. Importantly, molecular docking provides static predictions of ligand–protein spatial compatibility and does not establish direct target engagement, biochemical inhibition, binding kinetics, or functional MMP modulation. Because validation by re-docking of co-crystallized ligands was not performed, the docking results should be interpreted as hypothesis-generating structural predictions rather than quantitative evidence of ligand binding or inhibitory potency. To examine whether the predicted docking poses were compatible with the intrinsic conformational flexibility of the corresponding protein structures, coarse-grained molecular dynamics simulations were subsequently performed.

### 3.7. Comparative Physicochemical and In Silico Developability Assessment of LCC-10

**Figure 5:**
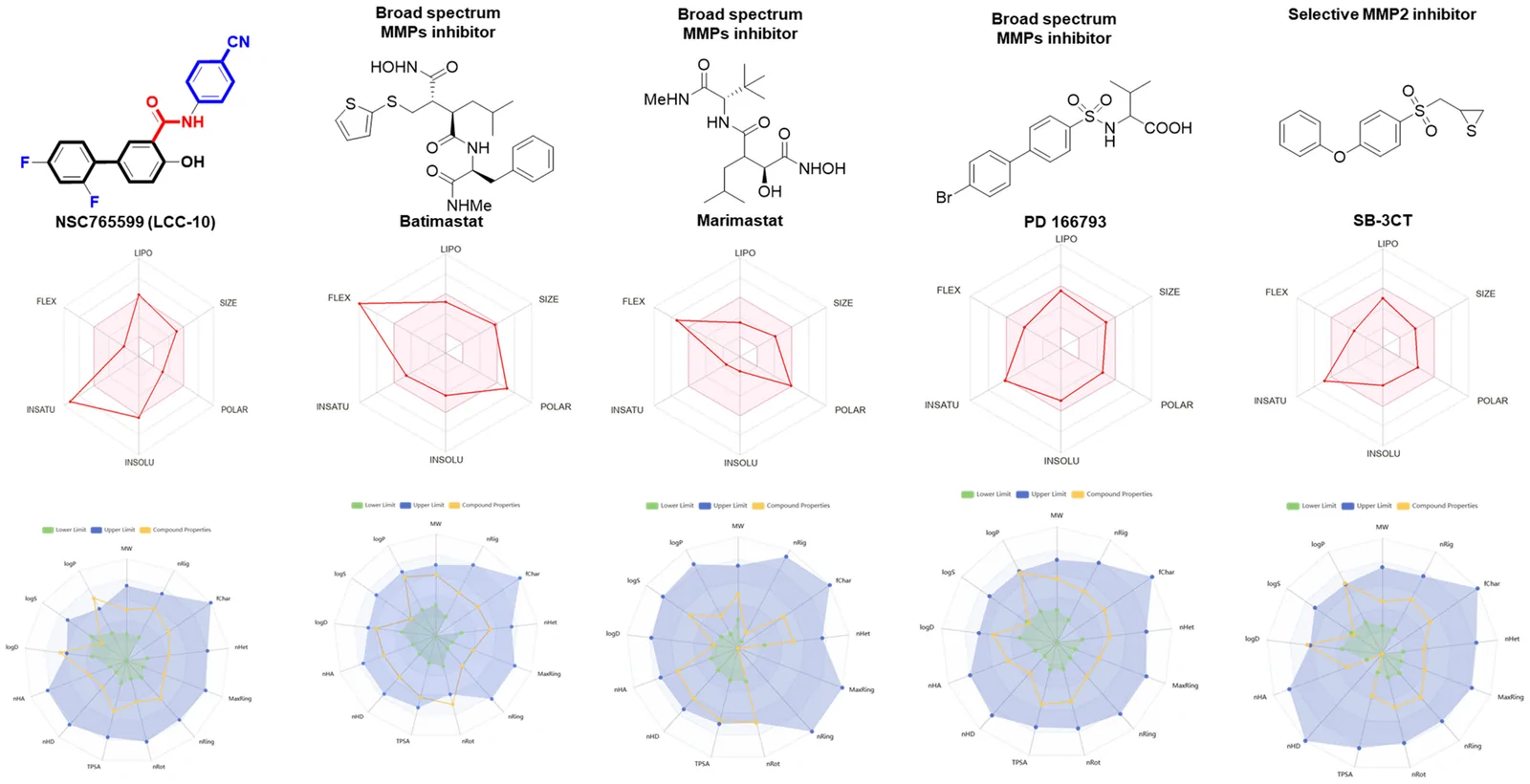
Comparative physicochemical and in silico developability assessment of LCC-10 and representative MMP inhibitors. Chemical structures of LCC-10, batimastat, marimastat, PD166793, and SB-3CT are shown in the upper panels. The middle panels show SwissADME bioavailability radar plots summarizing six physicochemical properties associated with oral drug-likeness (LIPO, SIZE, POLAR, INSOLU, INSATU, and FLEX). The lower panels summarize predicted pharmacokinetic, medicinal chemistry, and toxicity-related properties. These computational profiles are intended for comparative assessment and do not represent experimentally validated pharmacokinetic or safety properties. The complete ADMET prediction dataset is provided in Supplementary Table S4.

To further characterize the predicted developability profile of LCC-10, in silico physicochemical, pharmacokinetic, drug-likeness, and toxicity-related (ADMET) analyses were performed using SwissADME, ADMETLab 3.0, and pkCSM. The predicted properties of LCC-10 were systematically compared with those of representative MMP inhibitors. A summary of the principal physicochemical characteristics is presented in Figure 5, whereas the complete ADMET prediction dataset is provided in Supplementary Table S4. LCC-10 exhibited a molecular weight of 350.32 g/mol, a topological polar surface area (TPSA) of 73.12 Å^2^, four rotatable bonds, five hydrogen-bond acceptors, and two hydrogen-bond donors, indicating a relatively compact and conformationally restrained scaffold. Compared with batimastat and marimastat, LCC-10 possessed fewer rotatable bonds while retaining physicochemical properties consistent with commonly applied criteria for oral drug-likeness. SwissADME bioavailability radar analysis showed that the physicochemical profile of LCC-10 largely fell within the optimal physicochemical space defined by the model and substantially overlapped with those of the representative MMP inhibitors. Pharmacokinetic prediction suggested favorable gastrointestinal absorption and no predicted P-glycoprotein substrate liability. LCC-10 was also predicted to exhibit a relatively limited CYP interaction profile, with CYP2C9 emerging as the principal potential interaction among the evaluated major cytochrome P450 isoforms. Drug-likeness assessment indicated compliance with Lipinski’s Rule of Five and the Veber criteria, together with a predicted bioavailability score of 0.55, comparable to those of the representative MMP inhibitors [67]. In silicotoxicity assessmentdid notpredict Ames mutagenicity or major toxicity-related alerts among the evaluated endpoints, although a potential hERG-related liability was identified [68]. Collectively, these computational analyses suggest that LCC-10 possesses a physicochemical and predicted ADMET profile warranting further experimental evaluation. However, these in silico predictions do not establish in vivo pharmacokinetics or safety and require experimental validation.

### 3.8. CABS-Flex Analyses Support Comparable Conformational Flexibility of Representative MMP Structural Models

**Figure 6:**
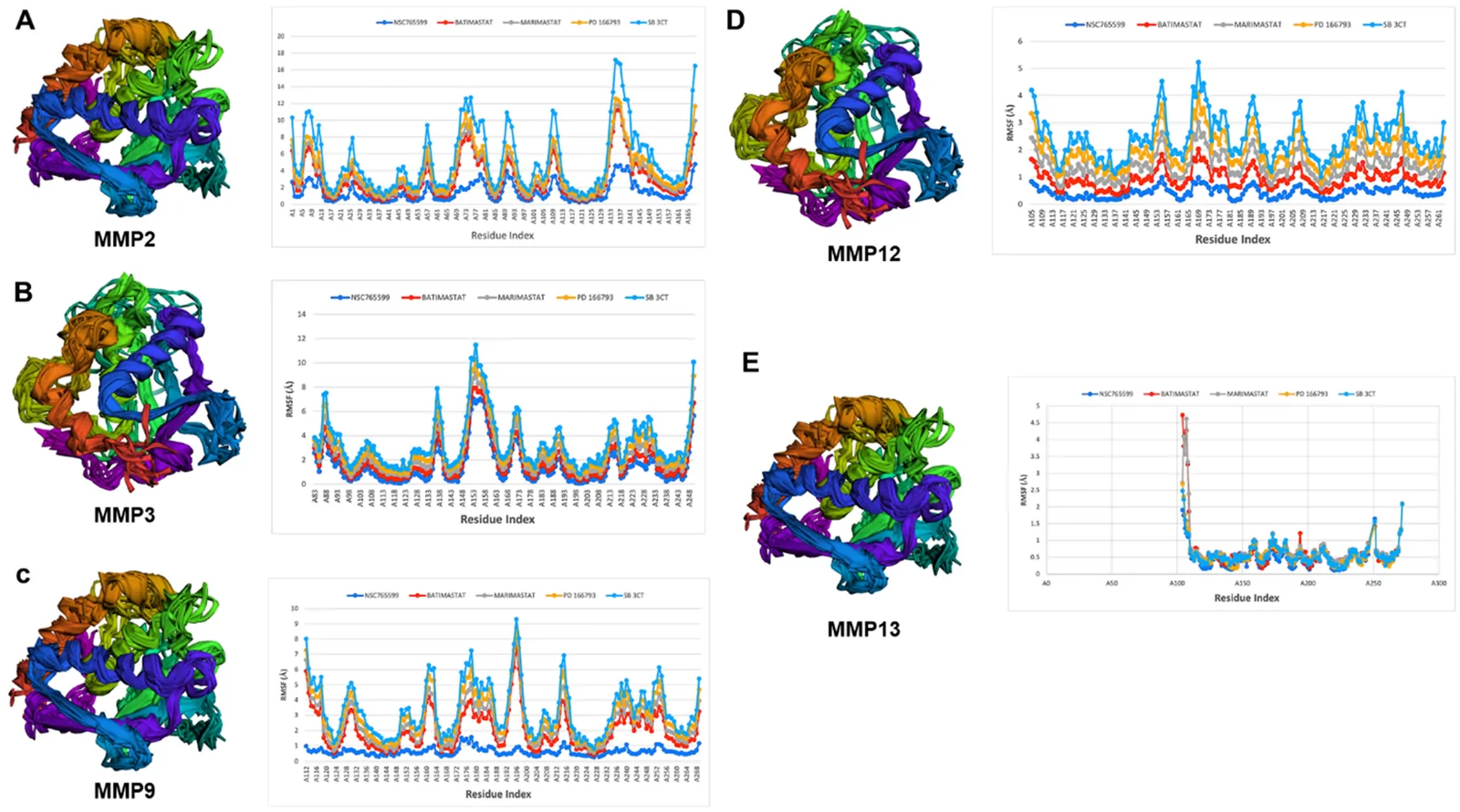
CABS-flex analyses show comparable conformational flexibility across representative MMP structural models. (**A**–**E**) CABS-flex conformational flexibility analyses of representative MMP2, MMP3, MMP9, MMP12, and MMP13 structural models selected from the docking analyses of LCC-10, batimastat, marimastat, PD166793, and SB-3CT. Left panels show superimposed backbone conformations sampled from the CABS-flex ensembles, whereas right panels show residue-level RMSF profiles. Broadly comparable RMSF patterns were observed across the analyzed structural models, supporting broadly similar predicted protein conformational flexibility. CABS-flex assesses protein flexibility and does not establish ligand retention, binding stability, biochemical inhibition, or direct target engagement.

To complement the molecular docking analysis, a coarse-grained conformational flexibility assessment was performed using the CABS-flex platform for representative MMP2, MMP3, MMP9, MMP12, and MMP13 structural models associated with the selected docking conditions for LCC-10 and the reference MMP inhibitors (Figure 6) [69]. Superposition of representative backbone conformations showed broadly similar global protein architectures across the analyzed structural ensembles. Residue-level root mean square fluctuation (RMSF) profiles indicated that conformational variability was predominantly localized to solvent-exposed loops and terminal regions, whereas residues within and surrounding the catalytic domains generally exhibited lower fluctuations [70]. Across the five representative MMPs, the CABS-flex-derived RMSF profiles of the structural models selected from the LCC-10 and reference-inhibitor docking analyses were broadly comparable. Localized differences in residue mobility were observed in several flexible regions; however, no consistent increase in global protein fluctuation was associated with LCC-10-related structural models. Notably, MMP13 exhibited a pronounced RMSF peak across the analyzed structural models, suggesting that this region represents an intrinsically flexible feature of the protein rather than a feature unique to the LCC-10-associated model [71]. Collectively, these analyses indicate that the MMP structural modelsassociated with the predicted LCC-10 docking poses exhibit conformational flexibility broadly comparable to that observed for the reference-inhibitor conditions. Importantly, CABS-flex evaluates coarse-grained protein conformational flexibility and does not quantify ligand-binding free energies, residence times, dissociation kinetics, or ligand retention within the binding pocket. [46]. Accordingly, these results should be interpreted solely as computational support for protein conformational compatibility and not as evidence of binding stability, direct target engagement, or biochemical inhibition. Together with the docking analyses, the CABS-flex results provide complementary structural context supporting further experimental evaluation of the predicted compatibility of LCC-10 with representative MMP catalytic domains.

### 3.9. Zebrafish Embryo Developmental Toxicity Assessment of LCC-10

**Figure 7:**
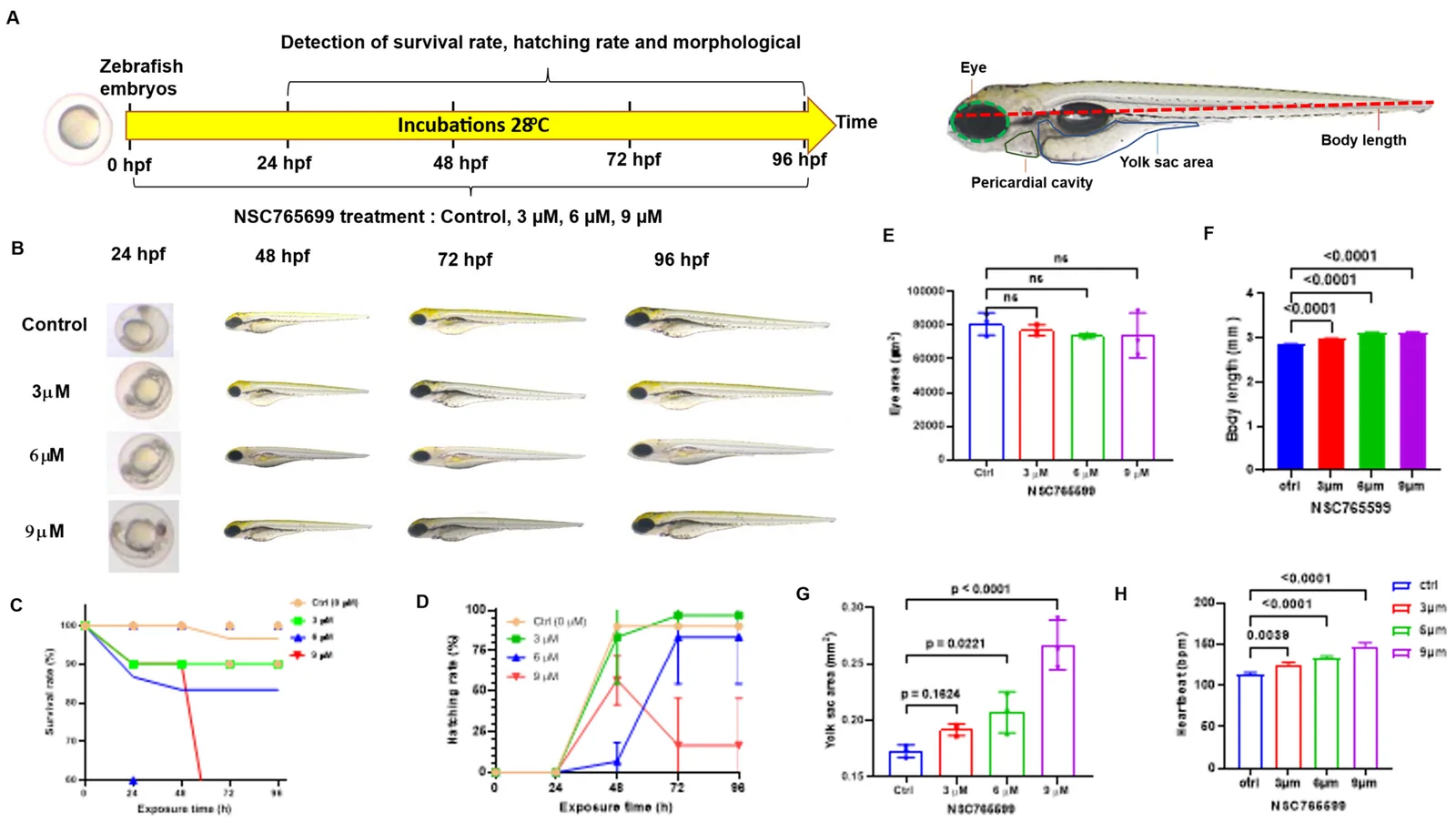
Zebrafish embryo developmental toxicity assessment of LCC-10. (**A**) Experimental design of the zebrafish embryo developmental toxicity assay. Embryos were exposed to vehicle control or LCC-10 (3, 6, or 9 μM) from 6 to 96 hpf, and survival, hatching, gross morphology, and developmental endpoints were evaluated during the exposure period. (**B**) Representative bright-field images of zebrafish embryos at 24, 48, 72, and 96 hpf following treatment with vehicle control or LCC-10. (**C**) Survival rates during continuous exposure. (**D**) Hatching rates during embryonic development. (**E**–**H**) Quantitative analyses of eye area (**E**), body length (**F**), yolk sac area (**G**), and heart rate (**H**) at 96 hpf. Data are presented as mean ± SD from three replicate wells per treatment group (10 embryos per well), with the replicate well considered the experimental unit. Statistical comparisons for morphometric endpoints (**E**–**H**) were performed using one-way ANOVA followed by Tukey’s multiple-comparison test. Exact *p*-values are shown; ns, not significant.

To complement the in silico ADMET predictions, zebrafish embryo developmental toxicity assays were performed to provide a preliminary in vivo assessment of LCC-10 tolerability [24]. Embryos were exposed to vehicle control or LCC-10 at concentrations of 3, 6, or 9 μM from 6 to 96 hpf, during which survival, hatching, gross morphology, and developmental endpoints were evaluated (Figure 7). Representative bright-field images showed generally preserved gross morphology across the treatment groups during embryonic development (Figure 7B). Survival remained relatively high at 3 and 6 μM, whereas exposure to 9 μM was associated with a more pronounced reduction in survival at later developmental stages (Figure 7C). Similarly, hatching progressed over time in the control and lower-concentration groups, whereas the 9 μM group exhibited a marked delay in hatching (Figure 7D).

Quantitative morphometric analyses at 96 hpf evaluated eye area, body length, yolk sac area, and heart rate (Figure 7E–H). Statistical comparisons among treatment groups were performed using one-way ANOVA followed by Tukey’s multiple-comparison test. For eye area (Figure 7E), no significant overall treatment effect was detected [F(3, 8) = 0.5525, *p* = 0.6606], and none of the LCC-10 treatment groups differed significantly from the control (3 μM, *p* = 0.5718; 6 μM, *p* = 0.2936; 9 μM, *p* = 0.3103). For body length (Figure 7F), a significant overall treatment effect was observed [F(3, 8) = 379.3, *p* < 0.0001], with significantly greater body length at 3, 6, and 9 μM than in the control group (all *p* < 0.0001). Yolk sac area (Figure 7G) also showed a significant overall treatment effect [F(3, 8) = 22.48, *p* = 0.0003]. Tukey’s post hoc analysis showed no significant difference at 3 μM (*p* = 0.1624), whereas significant increases were observed at 6 μM (*p* = 0.0221) and 9 μM (*p* < 0.0001) relative to the control, indicating a concentration-related alteration in this developmental endpoint. Heart rate (Figure 7H) likewise showed a significant overall treatment effect [F(3, 8) = 60.79, *p* < 0.0001], with significant increases at 3 μM (*p* = 0.0039), 6 μM (*p* < 0.0001), and 9 μM (*p* < 0.0001) compared with the control.

Collectively, these findings indicate that LCC-10 exposure was associated with measurable alterations in selected developmental endpoints, particularly yolk sac area and heart rate, while gross morphology remained generally preserved. The highest tested concentration (9 μM) was additionally associated with reduced survival and delayed hatching, indicating decreased developmental tolerability at higher exposure. Thus, the zebrafish embryo assay provides a preliminary assessment of developmental tolerability within the tested concentration range rather than establishing comprehensive in vivo safety. Although zebrafish embryos represent a useful vertebrate model for early developmental toxicity screening, additional mammalian pharmacokinetic and toxicological studies will be required to define the preclinical safety profile of LCC-10.

## 4. Discussion

Leukemia progression is increasingly recognized as a dynamic process driven not only by intrinsic genetic abnormalities but also by reciprocal interactions between leukemic cells and the bone marrow microenvironment. Although current therapeutic strategies predominantly target intracellular oncogenic signaling, accumulating evidence indicates that ECM remodeling contributes to leukemia cell survival, migration, therapeutic resistance, and niche maintenance. In the present study, we applied an integrated pharmacogenomic and structure-guided computational framework to investigate the biological context associated with the antileukemic activity of LCC-10. Rather than beginning with a predefined molecular target, our workflow integrated NCI-60 phenotypic profiling, DTP-COMPARE response similarity analysis, transcriptomic profiling, similarity-based target prediction, systems-level network analyses and functional enrichment analyses, molecular docking, coarse-grained conformational flexibility assessment, in silico developability prediction, and zebrafish embryo developmental toxicity evaluation. Across these complementary approaches, the findings prioritized an MMP-associated ECM regulatory network as a testable systems-level biological context associated with the pharmacological activity of LCC-10.

MMPs have traditionally been viewed as extracellular proteases responsible for matrix degradation; however, their biological functions extend substantially beyond ECM proteolysis. MMPs participate in cytokine activation, chemokine processing, angiogenesis, leukocyte trafficking, inflammatory signaling, and dynamic remodeling of the tumor microenvironment [72]. In hematological malignancies, dysregulated MMP activity has been implicated in bone marrow niche remodeling, leukemic cell dissemination, vascular invasion, immune modulation, and therapeutic resistance [73]. Consistent with these biological roles, transcriptomic profiling, functional enrichment, and systems-level network analyses in the present study collectively implicated coordinated ECM remodeling, inflammatory signaling, and extracellular proteolytic regulation rather than dependence on any individual MMP family member. Importantly, the CellMiner data used in this study represent baseline mRNA expression in untreated NCI-60 cells and therefore establish the presence and relative expression of candidate MMP-related genes rather than LCC-10-induced transcriptional regulation. Accordingly, the present findings are more appropriately interpreted as supporting an MMP-associated extracellular regulatory context than as demonstrating inhibition of any individual MMP by LCC-10.

Structure-based analyses provided an additional, but distinct, layer of support for this working hypothesis. Molecular docking indicated that LCC-10 could be accommodated within the catalytic domains of representative MMP isoforms, with predicted docking scores comparable to those obtained for the selected reference MMP inhibitors. These calculations therefore support the structural plausibilityof LCC-10 recognition by representative MMP catalytic pockets, but do not establish biochemical inhibition or direct target engagement. Complementary CABS-flex analyses showed that MMP structural models associated with the predicted LCC-10 docking poses exhibited residue-level conformational flexibility broadly comparable to that observed under the reference-inhibitor conditions. Importantly, CABS-flex evaluates coarse-grained protein conformational flexibility rather than ligand-binding free energy, residence time, dissociation kinetics, or ligand retention within the binding pocket. Thus, the docking and CABS-flex analyses should be interpreted collectively as computational support for structural and conformational compatibility, rather than as a demonstration of stable MMP binding or inhibition by LCC-10. Direct biochemical and target-engagement studies will therefore be required to determine whether the predicted MMP interactions occur experimentally.

The similarity-based target prediction also identified several cancer-associated intracellular proteins, including MTOR, CHEK1, and EGFR, as candidate targets of LCC-10. Because these proteins regulate cell survival and proliferative signaling, their prediction raises the possibility that intracellular mechanisms may contribute to the antiproliferative phenotype observed in the NCI-60 screen. The present data, however, do not establish functional inhibition of these kinases, nor do they determine whether intracellular signaling or extracellular protease-associated processes contribute more directly to LCC-10 activity. The MMP-associated network was prioritized in this study because multiple computational analyses converged on ECM remodeling and metalloprotease-related processes; nevertheless, alternative or overlapping mechanisms remain plausible and require experimental discrimination. Future studies integrating biochemical target assays, target-engagement measurements, and pathway-specific functional experiments will be necessary to distinguish among these candidate mechanisms.

Successful translation of an early-stage lead compound also depends on its physicochemical and preliminary safety characteristics. Comparative in silico analyses indicated that LCC-10 possesses physicochemical and drug-likeness properties generally comparable to those of the selected reference MMP inhibitors, together with pharmacokinetic and toxicity predictions that support further evaluation. These computational predictions were complemented by zebrafish embryo developmental toxicity testing. LCC-10 produced limited overt developmental effects at 3 and 6 μM, whereas exposure to 9 μM was associated with reduced survival and delayed hatching at later developmental stages. Major morphometric endpoints at 96 hpf were otherwise relatively preserved across the tested conditions. These findings therefore indicate a concentration-dependent preliminary tolerability profile rather than establishing comprehensive in vivo safety. Further mammalian pharmacokinetic, dose-ranging, and toxicological studies will be required before the developability of LCC-10 can be adequately defined.

The potential therapeutic positioning of LCC-10 in leukemia remains to be established. The present study was not designed to evaluate combination therapy, and no conclusions can therefore be drawn regarding interactions between LCC-10 and currently approved leukemia treatments. Nevertheless, the systems-level hypotheses generated here provide a rationale for future studies examining whether modulation of extracellular microenvironment-associated processes can complement established intracellularly targeted therapies. Such investigations should first establish the experimentally relevant molecular targets of LCC-10 and subsequently evaluate rational drug combinations in appropriate leukemia models.

A principal strength of this study is the integration of complementary phenotypic, pharmacogenomic, transcriptomic, network-based, structural, and preliminary experimental approaches within a unified hypothesis-generation framework. Rather than relying on a single prediction algorithm or target-specific screening strategy, the workflow enabled biological hypotheses emerging from one analytical layer to be examined in the context of complementary datasets. The convergence of several analyses on ECM remodeling and MMP-associated processes provides a rational basis for prioritizing these pathways for experimental testing. More broadly, this strategy illustrates how pharmacogenomic profiling and computational systems biology can be integrated to generate experimentally testable mechanistic hypotheses for phenotypically active compounds whose molecular targets remain incompletely characterized.

Several limitations should be acknowledged. First, the proposed MMP-associated ECM regulatory network represents a computationally prioritized working hypothesis rather than an experimentally established mechanism of action. Direct validation will require biochemical assays using recombinant MMPs, together with orthogonal target-engagement approaches and functional assays relevant to ECM remodeling, invasion, and transendothelial migration. Such experiments will be necessary to determine whether MMP-associated processes contribute causally to the antiproliferative phenotype of LCC-10 or instead represent one component of a broader pharmacological response. Second, the CABS-flex analyses assess coarse-grained protein conformational flexibility and cannot establish ligand-binding energetics, residence time, or biochemical inhibition. Third, the zebrafish embryo assay provides only a preliminary assessment of developmental tolerability, with adverse effects on survival and hatching becoming apparent at the highest tested concentration. Additional pharmacokinetic, efficacy, and toxicological studies in mammalian models will therefore be required. Finally, validation in additional leukemia models and primary patient-derived samples will be important to establish the generalizability and disease relevance of the proposed biological framework.

An important limitation of the present analysis is that the NCI-60 screening platform primarily measures tumor-cell growth responses under monoculture conditions. In contrast, many MMP-associated functions—including extracellular proteolysis, invasion, and microenvironmental remodeling—depend on extracellular matrix and stromal contexts that are not represented in the NCI-60 assay. Therefore, the NCI-60 growth-inhibition phenotype cannot directly establish whether MMP-associated extracellular processes contribute functionally to the activity of LCC-10. Future studies should incorporate post-treatment transcriptomic profiling, such as RNA-seq following LCC-10 exposure, to determine whether MMP-associated, ECM-remodeling, inflammatory, or other candidate pathways are dynamically altered by treatment. In addition, zebrafish leukemia xenograft models could provide a complementary in vivo platform for examining whether LCC-10 affects leukemic-cell migration and dissemination. Together with biochemical and target-engagement assays, these approaches will help experimentally test the systems-level hypotheses generated in the present study.

**Figure 8:**
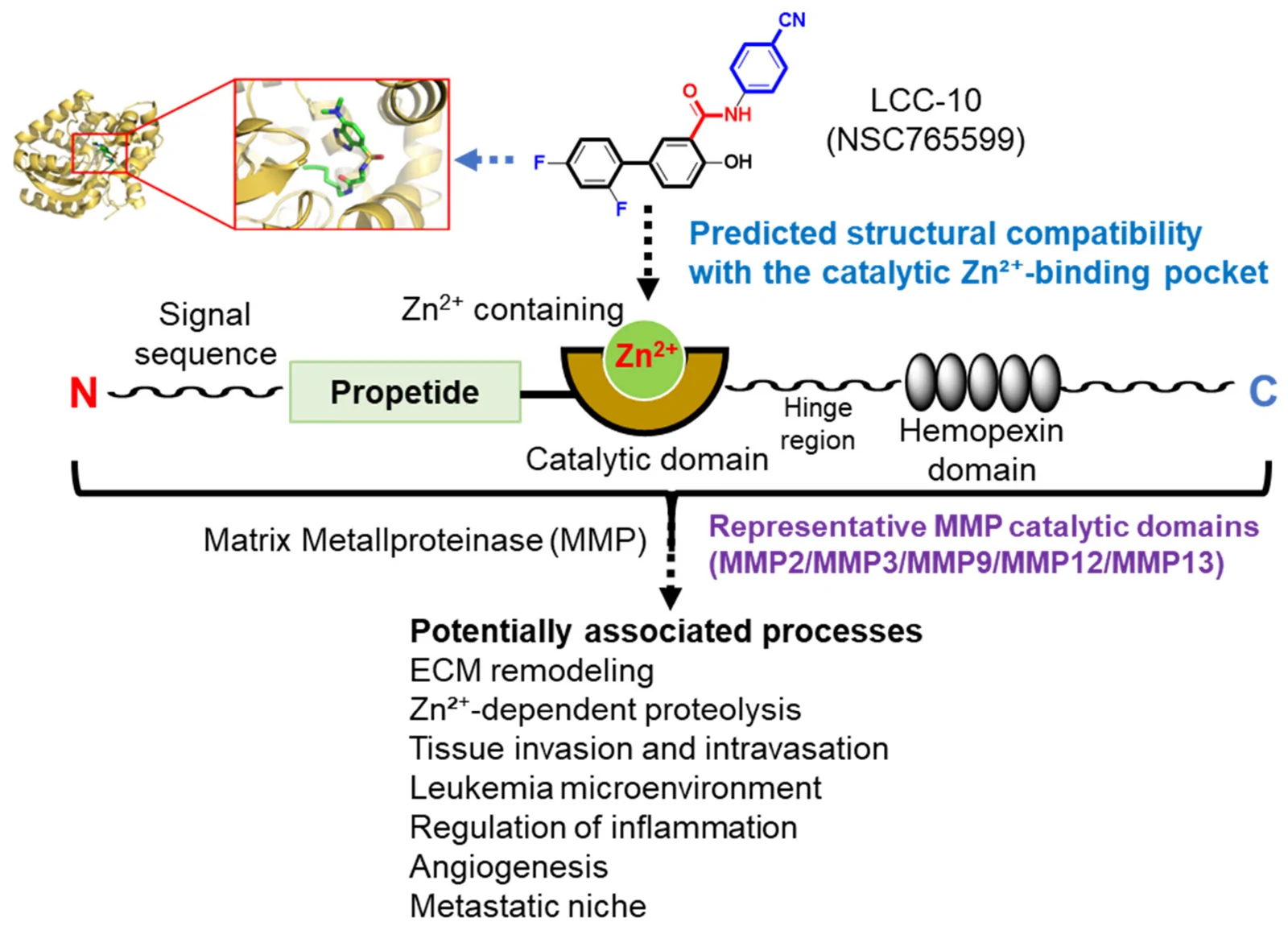
Proposed systems-level working model for the biological context associated with the antileukemic activity of LCC-10. Integrated pharmacogenomic, transcriptomic, systems-level network, and structure-based analyses collectively prioritized an MMP-associated ECM regulatory network as a testable working hypothesis. Structure-guided analyses support the predicted structural compatibility of LCC-10 with representative MMP catalytic domains, whereas transcriptomic and systems-level analyses implicate coordinated ECM remodeling, extracellular proteolysis, inflammatory signaling, and microenvironment-associated processes. The proposed model represents a hypothesis-generating framework and does not imply experimentally confirmed MMP binding, enzymatic inhibition, or a validated mechanism of action.

The integrated findings of the present study are summarized in the proposed systems-level working model shown in Figure 8, which highlights an MMP-associated ECM regulatory network as a testable biological context potentially associated with the antileukemic activity of LCC-10. Accordingly, the present study should be regarded as a systems-level, hypothesis-generating investigation that establishes a foundation for subsequent biochemical, target-engagement, and functional validation.

## 5. Conclusions

In conclusion, this study establishes an integrated pharmacogenomic and structure-guided computational framework for investigating the biological context associated with the antileukemic activity of LCC-10. By combining NCI-60 phenotypic profiling, pharmacogenomic response similarity analysis, transcriptomic profiling, similarity-based target prediction, functional enrichment and systems-level network analysis, molecular docking, coarse-grained conformational flexibility assessment, comparative in silico ADMET evaluation, and zebrafish embryo developmental toxicity testing, multiple complementary analytical approaches collectively prioritized an MMP-associated ECM regulatory network as a testable systems-level hypothesis associated with LCC-10 activity. Structure-based analyses supported the predicted structural compatibility of LCC-10 with representative MMP catalytic domains, whereas the zebrafish embryo studies provided a preliminary assessment of developmental tolerability within the tested concentration range. Rather than establishing a definitive molecular target or mechanism of action, the present findings provide a hypothesis-generating framework linking LCC-10 to MMP-associated ECM remodeling and leukemia microenvironment-related processes. These results provide a rationale for prioritizing representative MMP family members for subsequent biochemical, target-engagement, and functional validation and illustrate the utility of integrating pharmacogenomics, systems biology, and structure-guided modeling for mechanism-oriented anticancer drug discovery. Overall, this study provides a rational foundation for future experimental validation of the molecular and functional determinants underlying the antileukemic activity of LCC-10.

## Data Availability

The datasets used and analyzed during the current study are available from the corresponding author upon reasonable request.

## Supplementary Materials

The following supporting information can be downloaded at: https://www.mdpi.com/article/10.3390/cells15171610/s1, Figure S1: Complete NCI-60 pharmacological response profiles of LCC-10 (NSC765599). Upper panels show the single-dose (10 μM) growth inhibition profile across the NCI-60 Human Tumor Cell Line Screen. Lower panels present the corresponding five-dose concentration–response profiles and derived pharmacological response parameters (GI50, TGI, LC50, and IC50). The leukemia-associated results shown in Figure 1 were derived from this complete screening dataset.; Table S1: Pharmacogenomic response similarity analysis of NSC765599 using DTP-COMPARE. Pearson’s correlation coefficient (r) and common cell line count (CCLC) were used to quantify similarity between growth inhibition profiles across the NCI-60 panel. Synthetic compounds and clinically used anticancer agents are listed for comparison.; Table S2: Predicted protein targets of LCC-10 (NSC765599) identified using SwissTargetPrediction. Predicted protein targets are grouped according to functional class, including proteases, receptor tyrosine kinases, serine/threonine kinases, enzymes, and secreted proteins. UniProt and ChEMBL identifiers are provided to facilitate target annotation and downstream structure-guided computational analyses.; Table S3: Docking setup, re-docking validation, AutoDock Vina docking scores, and representative ligand–protein interactions of LCC-10 (NSC765599) and reference MMP inhibitors across five representative MMP isoforms. Part A summarizes the receptor structures, docking-grid parameters, AutoDock Vina exhaustiveness setting, and re-docking RMSD values. Part B reports the predicted Vina docking scores and representative interactions identified from the top-ranked docking poses. Docking scores are computational predictions and should not be interpreted as experimental binding affinities, biochemical inhibition constants, or direct target engagement.; Table S4: Comprehensive in silico physicochemical, pharmacokinetic, medicinal chemistry, and toxicity-related (ADMET) prediction profiles of LCC-10 and representative matrix metalloproteinase inhibitors. Predictions were generated using SwissADME, ADMETLab 3.0, and pkCSM. Binary outputs (Yes/No) indicate model-based classifications, whereas numerical values represent predicted physicochemical parameters, probabilities, or risk scores.
