# Supplementary material for: Integrated Pharmacogenomic and Structure-Guided Analyses Link LCC-10 (NSC765599) to an MMP-Associated Extracellular Matrix Regulatory Network in Leukemia
Source: Cells. 2026 Sep 4;15(17):1610. doi: 10.3390/cells15171610 (PMC13564721; doi:10.3390/cells15171610)
Supplement: Supplementary file 1 [file cells-15-01610-s001.zip › Supplementary Figure S1_20260804_final.pdf]

Supplementary Figure S1. Complete NCI-60 pharmacological response profiles of LCC-10 (NSC765599). Upper panels show the single-dose (10 μM) growth inhibition profile across the NCI-60 Human Tumor Cell Line Screen. Lower panels present the corresponding five-dose concentration–response profiles and derived pharmacological response parameters (GI_{50}, TGI, LC_{50}, and IC_{50}). The leukemia-associated results shown in **Figure 1** were derived from this complete screening dataset.

NSC765599 (one dose assay):

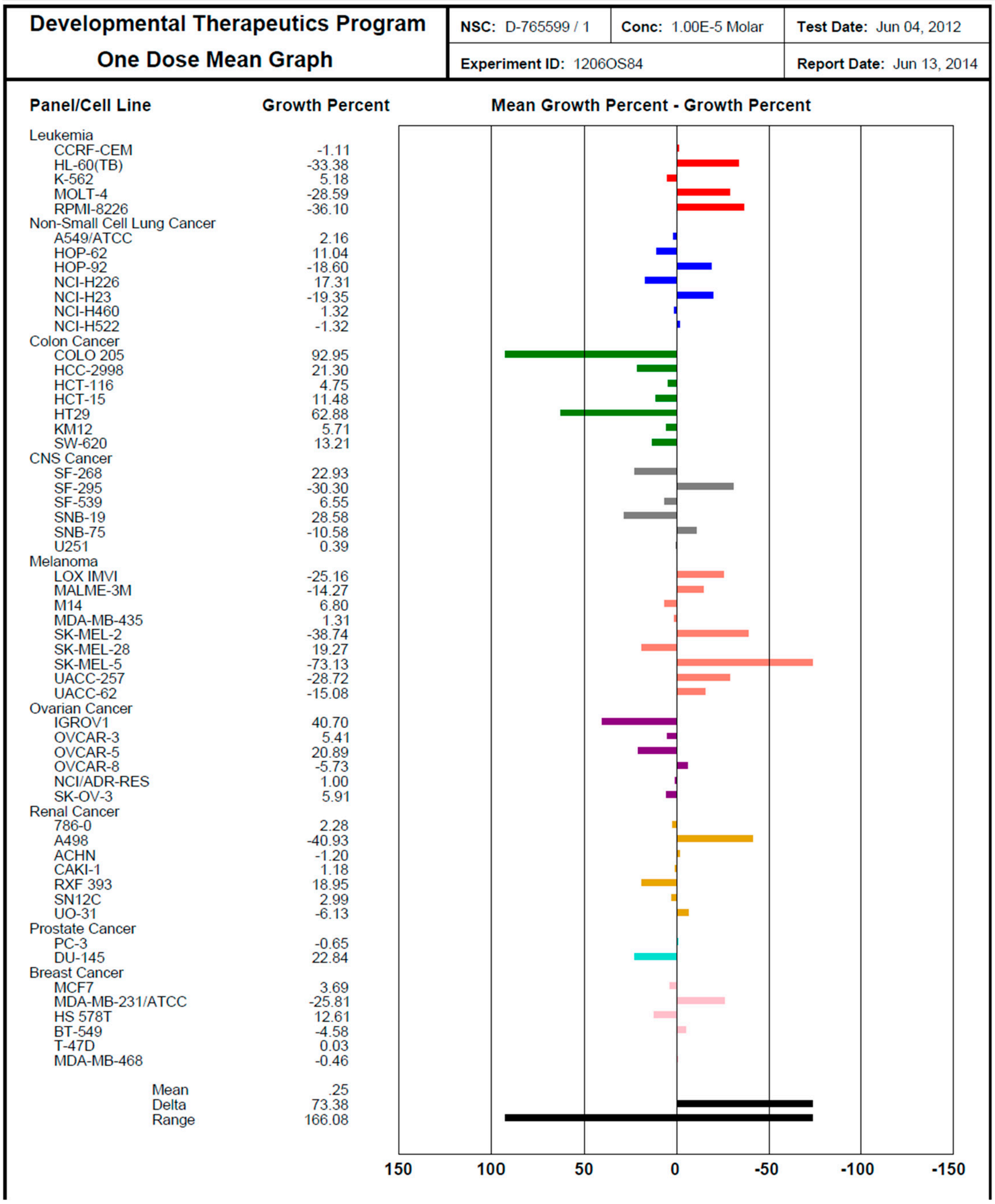

NSC765599 (five dose assay):

National Cancer Institute Developmental Therapeutics Program
In-Vitro Testing Results

NSC : D - 765599 / 1	Experiment ID : 1207NS08	Test Type : 08	Units : Molar
Report Date : June 06, 2025	Test Date : July 16, 2012	QNS :	MC :
COMI :	Stain Reagent : SRB Dual-Pass Related	SSPL :	

Log10 Concentration

Panel/Cell Line	Time Zero	Ctrl	Mean Optical Densities -8.0	-7.0	-6.0	-5.0	-4.0	Percent Growth -8.0	-7.0	-6.0	-5.0	-4.0	GI50	TGI	LC50
**Leukemia**															
CCRF-CEM	0.627	2.587	2.559	2.435	1.458	0.538	0.362	99	92	42	-14	-42	7.03E-7	5.60E-6	> 1.00E-4
HL-60(TB)	0.895	2.825	2.859	2.785	1.581	0.469	0.734	102	98	36	-48	-18	5.86E-7	2.68E-6	> 1.00E-4
K-562	0.240	1.981	2.040	2.005	0.884	0.273	0.229	103	101	37	2	-5	6.28E-7	1.91E-5	> 1.00E-4
MOLT-4	0.664	2.331	2.478	2.458	1.466	0.381	0.441	109	108	48	-43	-34	9.28E-7	3.39E-6	> 1.00E-4
RPMI-8226	1.272	2.819	2.738	2.569	1.248	0.703	0.831	95	84	-2	-45	-35	2.48E-7	9.51E-7	> 1.00E-4
SR	0.221	0.583	0.566	0.541	0.486	0.207	0.209	95	88	73	-7	-6	1.95E-6	8.27E-6	> 1.00E-4
**Non-Small Cell Lung Cancer**															
A549/ATCC	0.400	1.789	1.718	1.684	1.108	0.419	0.200	95	92	51	1	-50	1.05E-6	1.06E-5	9.94E-5
HOP-62	0.410	1.247	1.280	1.307	1.180	0.356	0.091	104	107	92	-13	-78	2.51E-6	7.48E-6	3.70E-5
HOP-92	1.410	1.799	1.742	1.743	1.138	0.989	0.600	85	86	-19	-30	-57	2.19E-7	6.55E-7	5.37E-5
NCI-H226	0.904	2.393	2.410	2.452	1.864	0.957	0.537	101	104	64	4	-41	1.73E-6	1.20E-5	> 1.00E-4
NCI-H23	0.821	2.254	2.239	2.241	1.062	0.618	0.405	99	99	17	-25	-51	3.95E-7	2.54E-6	9.42E-5
NCI-H322M	0.815	1.581	1.581	1.601	1.263	0.687	0.511	100	103	58	-16	-37	1.30E-6	6.13E-6	> 1.00E-4
NCI-H460	0.264	2.668	2.659	2.615	1.094	0.289	0.176	100	98	35	1	-33	5.69E-7	1.07E-5	> 1.00E-4
**Colon Cancer**															
COLO 205	0.380	1.577	1.631	1.665	1.625	0.632	0.043	105	107	104	21	-89	4.48E-6	1.56E-5	4.44E-5
HCC-2998	1.199	3.210	3.226	3.229	3.212	0.831	0.123	101	101	100	-31	-90	2.42E-6	5.83E-6	2.12E-5
HCT-116	0.187	1.689	1.764	1.704	0.975	0.069	0.009	105	101	52	-63	-95	1.05E-6	2.84E-6	7.70E-6
HCT-15	0.160	1.787	1.699	1.684	1.046	0.193	0.106	95	94	54	2	-34	1.22E-6	1.14E-5	> 1.00E-4
HT29	0.303	1.466	1.502	1.582	1.560	0.302	0.035	103	110	108	0	-88	3.43E-6	9.93E-6	3.66E-5
KM12	0.409	2.108	2.081	2.180	1.618	0.412	0.176	98	104	71	0	-57	1.99E-6	1.01E-5	7.55E-5
SW-620	0.247	2.016	1.954	1.911	1.494	0.365	0.143	96	94	71	7	-42	2.10E-6	1.37E-5	> 1.00E-4
**CNS Cancer**															
SF-268	0.562	1.886	1.900	1.960	1.453	0.773	0.422	101	106	67	16	-25	2.17E-6	2.46E-5	> 1.00E-4
SF-539	0.493	2.259	2.240	2.163	1.612	0.674	0.175	99	95	63	10	-65	1.78E-6	1.37E-5	6.38E-5
SNB-19	0.824	2.209	2.112	2.084	1.299	0.958	0.464	93	91	34	10	-44	5.29E-7	1.52E-5	> 1.00E-4
SNB-75	1.227	2.202	2.026	1.993	1.796	1.219	0.628	82	79	58	0	-49	1.39E-6	9.75E-6	> 1.00E-4
U251	0.496	2.087	2.028	1.953	1.128	0.410	0.068	96	92	40	-17	-86	6.33E-7	4.97E-6	2.97E-5
**Melanoma**															
LOX IMVI	0.715	3.057	3.064	3.162	1.579	0.478	0.106	100	104	37	-33	-85	6.40E-7	3.36E-6	2.10E-5
MALME-3M	0.563	1.007	0.994	0.992	0.788	0.322	0.310	97	97	51	-43	-45	1.02E-6	3.48E-6	> 1.00E-4
M14	0.445	1.755	1.759	1.763	1.256	0.343	0.219	100	101	62	-23	-51	1.38E-6	5.37E-6	9.37E-5
MDA-MB-435	0.376	1.458	1.422	1.361	1.036	0.255	0.149	97	91	61	-32	-60	1.31E-6	4.50E-6	4.27E-5
SK-MEL-2	1.033	1.849	1.926	1.942	1.167	0.460	0.319	109	111	16	-55	-69	4.43E-7	1.69E-6	8.39E-6
SK-MEL-28	0.539	1.402	1.441	1.357	0.945	0.531	0.379	105	95	47	-2	-30	8.65E-7	9.28E-6	> 1.00E-4
SK-MEL-5	0.676	2.965	2.967	2.911	0.886	0.116	0.116	100	98	9	-83	-83	3.45E-7	1.26E-6	4.39E-6
UACC-257	0.900	1.946	1.866	1.836	1.383	0.709	0.676	92	89	46	-21	-25	8.15E-7	4.84E-6	> 1.00E-4
UACC-62	1.049	2.859	2.728	2.767	1.153	0.428	0.175	93	95	6	-59	-83	3.19E-7	1.22E-6	7.21E-6
**Ovarian Cancer**															
IGROV1	0.638	1.741	1.914	1.836	1.162	0.654	0.325	116	109	47	1	-49	9.09E-7	1.07E-5	> 1.00E-4
OVCAR-3	0.485	1.539	1.592	1.611	0.888	0.591	0.272	105	107	38	10	-44	6.74E-7	1.53E-5	> 1.00E-4
OVCAR-4	0.769	1.594	1.597	1.535	1.153	0.853	0.553	100	93	47	10	-28	8.41E-7	1.85E-5	> 1.00E-4
OVCAR-5	0.434	1.335	1.346	1.312	1.268	0.753	0.472	101	97	93	35	4	5.55E-6	> 1.00E-4	> 1.00E-4
OVCAR-8	0.417	1.660	1.656	1.668	1.209	0.419	0.263	100	101	64	0	-37	1.64E-6	1.01E-5	> 1.00E-4
NCI/ADR-RES	0.748	2.288	2.313	2.375	1.390	0.748	0.461	102	106	42	0	-38	7.41E-7	9.96E-6	> 1.00E-4
SK-OV-3	0.592	1.272	1.315	1.300	1.172	0.613	0.360	106	104	85	3	-39	2.68E-6	1.18E-5	> 1.00E-4
**Renal Cancer**															
786-0	0.838	2.794	2.773	2.829	2.688	0.722	0.216	99	102	95	-14	-74	2.58E-6	7.45E-6	3.97E-5
A498	1.396	2.083	1.946	1.887	1.535	0.692	0.718	80	72	20	-50	-49	2.63E-7	1.93E-6	.
ACHN	0.259	1.512	1.520	1.501	1.052	0.357	0.151	101	99	63	8	-42	1.73E-6	1.44E-5	> 1.00E-4
CAKI-1	1.099	2.732	2.632	2.562	1.758	0.815	0.556	94	90	40	-26	-49	6.37E-7	4.07E-6	> 1.00E-4
RXF 393	0.885	1.264	1.278	1.301	1.247	0.709	0.222	104	110	96	-20	-75	2.48E-6	6.72E-6	3.52E-5
SN12C	0.958	2.860	2.746	2.685	1.358	0.861	0.547	94	91	21	-10	-43	3.84E-7	4.73E-6	> 1.00E-4
TK-10	0.779	1.374	1.326	1.317	1.149	0.685	0.363	92	90	62	-12	-53	1.46E-6	6.87E-6	8.27E-5
UO-31	0.680	1.939	1.877	1.955	1.355	0.612	0.080	95	101	54	-10	-88	1.14E-6	6.95E-6	3.24E-5
**Prostate Cancer**															
PC-3	0.518	1.972	1.927	1.896	1.380	0.407	0.173	97	95	59	-21	-67	1.30E-6	5.43E-6	4.28E-5
DU-145	0.389	1.569	1.660	1.654	1.184	0.572	0.286	108	107	67	15	-26	2.16E-6	2.34E-5	> 1.00E-4
**Breast Cancer**															
MCF7	0.424	2.312	2.115	2.144	0.991	0.483	0.334	90	91	30	3	-21	4.71E-7	1.34E-5	> 1.00E-4
MDA-MB-231/ATCC	0.677	1.385	1.372	1.380	1.170	0.453	0.291	98	99	70	-33	-57	1.55E-6	4.76E-6	5.05E-5
HS 578T	0.912	1.899	1.862	1.865	1.476	0.877	0.601	96	97	57	-4	-34	1.31E-6	8.65E-6	> 1.00E-4
BT-549	0.746	1.813	1.767	1.874	1.316	0.607	0.268	96	106	53	-19	-64	1.11E-6	5.50E-6	4.88E-5
T-47D	0.684	1.645	1.629	1.652	0.905	0.662	0.595	98	101	23	-3	-13	4.49E-7	7.49E-6	> 1.00E-4
MDA-MB-468	0.767	1.679	1.618	1.603	0.999	0.660	0.510	93	92	25	-14	-34	4.25E-7	4.42E-6	> 1.00E-4

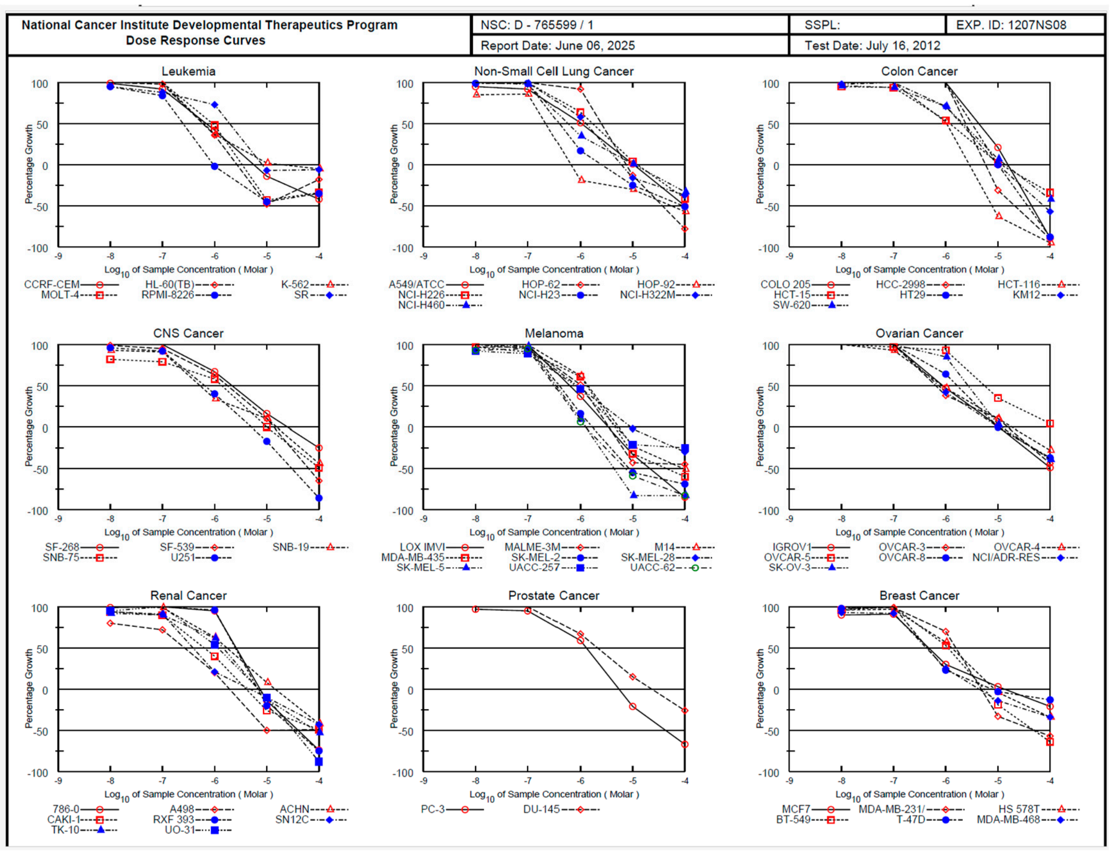
National Cancer Institute Developmental Therapeutics Program
Dose Response Curves
NSC: D - 765599 / 1
Report Date: June 06, 2025
SSPL:
EXP. ID: 1207NS08
Test Date: July 16, 2012
Leukemia
Non-Small Cell Lung Cancer
Colon Cancer
CNS Cancer
Melanoma
Ovarian Cancer
Renal Cancer
Prostate Cancer
Breast Cancer
Percentage Growth
Log10 of Sample Concentration (Molar)
CCRF-CEM
HL-60(TB)
K-562
MOLT-4
RPMI-8226
SR
A549/ATCC
HOP-62
HOP-92
NCI-H226
NCI-H23
NCI-H322M
NCI-H460
COLO 205
HCC-2998
HCT-116
HCT-15
HT29
KM12
SW-620
SF-268
SF-539
SNB-19
SNB-75
U251
LOX IMVI
MALME-3M
M14
MDA-MB-435
SK-MEL-2
SK-MEL-28
SK-MEL-5
UACC-257
UACC-62
IGROV1
OVCAR-3
OVCAR-4
OVCAR-5
OVCAR-8
NCI/ADR-RES
SK-OV-3
786-0
A498
ACHN
CAKI-1
RXF 393
SN12C
TK-10
UO-31
PC-3
DU-145
MCF7
MDA-MB-231/
HS 578T
BT-549
T-47D
MDA-MB-468

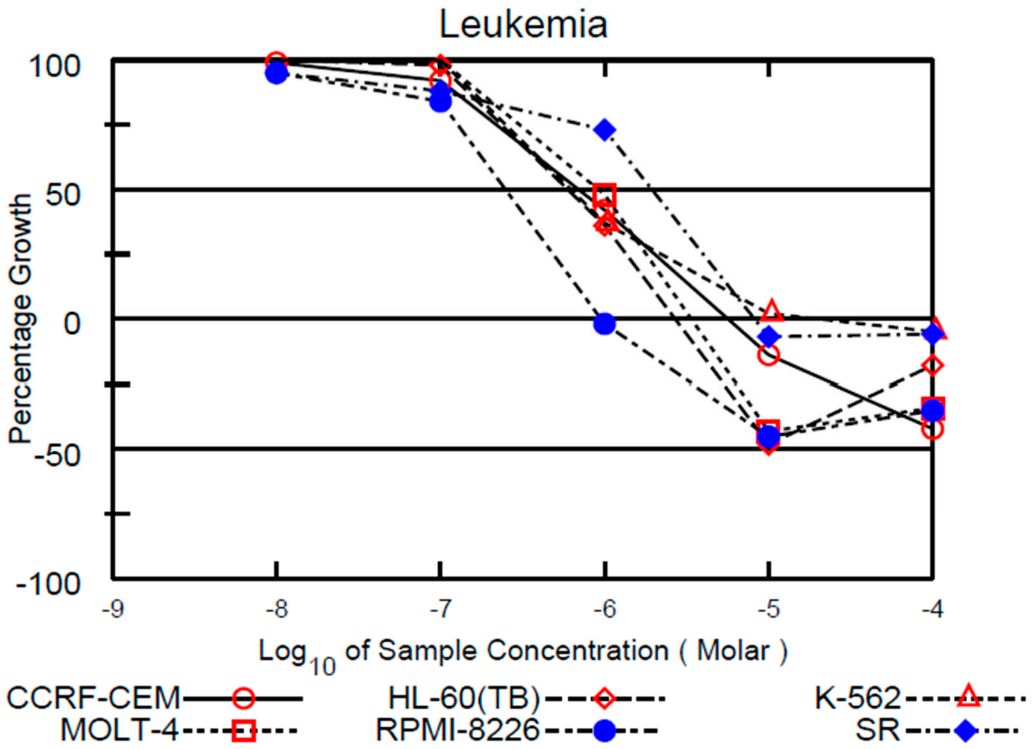
Leukemia
100
50
0
-50
-100
Percentage Growth
-9
-8
-7
-6
-5
-4
Log10 of Sample Concentration (Molar)
CCRF-CEM
HL-60(TB)
K-562
MOLT-4
RPMI-8226
SR

National Cancer Institute Developmental Therapeutics Program
Mean Graphs

NSC : D - 765599/1	Units :Molar	SSPL :	EXP. ID :1207NS08
Report Date :June 06, 2025		Test Date :July 16, 2012	

Panel/Cell Line	Log_{10}GI50	Log_{10}TGI	Log_{10}LC50
**Leukemia**			
CCRF-CEM	-6.15	-5.25	> -4.00
HL-60(TB)	-6.23	-5.57	> -4.00
K-562	-6.20	-4.72	> -4.00
MOLT-4	-6.03	-5.47	> -4.00
RPMI-8226	-6.61	-6.02	> -4.00
SR	-5.71	-5.08	> -4.00
**Non-Small Cell Lung Cancer**			
A549/ATCC	-5.98	-4.97	-4.00
HOP-62	-5.60	-5.13	-4.43
HOP-92	-6.66	-6.18	-4.27
NCI-H226	-5.76	-4.92	> -4.00
NCI-H23	-6.40	-5.60	-4.03
NCI-H322M	-5.89	-5.21	> -4.00
NCI-H460	-6.24	-4.97	> -4.00
**Colon Cancer**			
COLO 205	-5.35	-4.81	-4.35
HCC-2998	-5.62	-5.23	-4.67
HCT-116	-5.98	-5.55	-5.11
HCT-15	-5.92	-4.94	> -4.00
HT29	-5.46	-5.00	-4.44
KM12	-5.70	-5.00	-4.12
SW-620	-5.68	-4.86	> -4.00
**CNS Cancer**			
SF-268	-5.66	-4.61	> -4.00
SF-539	-5.75	-4.86	-4.20
SNB-19	-6.28	-4.82	> -4.00
SNB-75	-5.86	-5.01	> -4.00
U251	-6.20	-5.30	-4.53
**Melanoma**			
LOX IMVI	-6.19	-5.47	-4.68
MALME-3M	-5.99	-5.46	> -4.00
M14	-5.86	-5.27	-4.03
MDA-MB-435	-5.88	-5.35	-4.37
SK-MEL-2	-6.35	-5.77	-5.08
SK-MEL-28	-6.06	-5.03	> -4.00
SK-MEL-5	-6.46	-5.90	-5.36
UACC-257	-6.09	-5.32	> -4.00
UACC-62	-6.50	-5.91	-5.14
**Ovarian Cancer**			
IGROV1	-6.04	-4.97	> -4.00
OVCAR-3	-6.17	-4.81	> -4.00
OVCAR-4	-6.08	-4.73	> -4.00
OVCAR-5	-5.26	> -4.00	> -4.00
OVCAR-8	-5.78	-5.00	> -4.00
NCI/ADR-RES	-6.13	-5.00	> -4.00
SK-OV-3	-5.57	-4.93	> -4.00
**Renal Cancer**			
786-0	-5.59	-5.13	-4.40
A498	-6.58	-5.71	
ACHN	-5.76	-4.84	> -4.00
CAKI-1	-6.20	-5.39	> -4.00
RXF 393	-5.61	-5.17	-4.45
SN12C	-6.42	-5.33	> -4.00
TK-10	-5.84	-5.16	-4.08
UO-31	-5.94	-5.16	-4.49
**Prostate Cancer**			
PC-3	-5.88	-5.27	-4.37
DU-145	-5.66	-4.63	> -4.00
**Breast Cancer**			
MCF7	-6.33	-4.87	> -4.00
MDA-MB-231/ATCC	-5.81	-5.32	-4.30
HS 578T	-5.88	-5.06	> -4.00
BT-549	-5.95	-5.26	-4.31
T-47D	-6.35	-5.13	> -4.00
MDA-MB-468	-6.37	-5.35	> -4.00

National Cancer Institute Developmental Therapeutics Program
Dose Response Curves

NSC : D - 765599/1	SSPL:	EXP. ID:1207NS08
Report Date:June 06, 2025	Test Date:July 16, 2012	

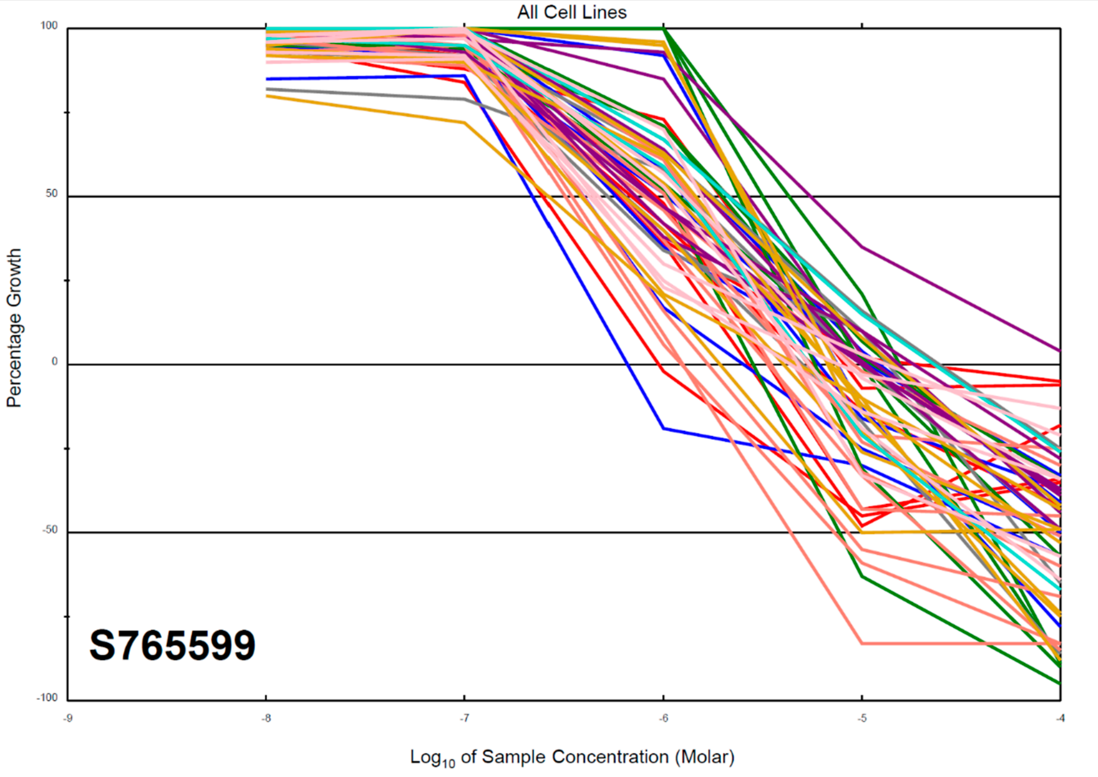

National Cancer Institute Developmental Therapeutics Program<br>Waterfall Graph GI50	NSC : D - 765599/1	Units :Molar	SSPL :	EXP. ID :1207NS08
	Report Date :June 06, 2025		Test Date :July 16, 2012	

Panel	Cell Name	Hollow Fiber	GI50
Non-Small Cell Lung Cancer	HOP-92		-6.66
Leukemia	RPMI-8226		-6.61
Renal Cancer	A498		-6.58
Melanoma	UACC-62	*	-6.50
Melanoma	SK-MEL-5		-6.46
Renal Cancer	SN12C		-6.42
Non-Small Cell Lung Cancer	NCI-H23	*	-6.40
Breast Cancer	MDA-MB-468		-6.37
Melanoma	SK-MEL-2		-6.35
Breast Cancer	T-47D		-6.35
Breast Cancer	MCF7		-6.33
CNS Cancer	SNB-19		-6.28
Non-Small Cell Lung Cancer	NCI-H460		-6.24
Leukemia	HL-60(TB)		-6.23
Leukemia	K-562		-6.20
CNS Cancer	U251	*	-6.20
Renal Cancer	CAKI-1		-6.20
Melanoma	LOX IMVI	*	-6.19
Ovarian Cancer	OVCAR-3	*	-6.17
Leukemia	CCRF-CEM		-6.15
Ovarian Cancer	NCI/ADR-RES		-6.13
Melanoma	UACC-257		-6.09
Ovarian Cancer	OVCAR-4		-6.08
Melanoma	SK-MEL-28		-6.06
Ovarian Cancer	IGROV1		-6.04
Leukemia	MOLT-4		-6.03
Melanoma	MALME-3M		-5.99
Non-Small Cell Lung Cancer	A549/ATCC		-5.98
Colon Cancer	HCT-116		-5.98
Breast Cancer	BT-549		-5.95
Renal Cancer	UO-31		-5.94
Colon Cancer	HCT-15		-5.92
Non-Small Cell Lung Cancer	NCI-H322M		-5.89
Prostate Cancer	PC-3		-5.88
Breast Cancer	HS 578T		-5.88
Melanoma	MDA-MB-435	*	-5.88
Melanoma	M14		-5.86
CNS Cancer	SNB-75		-5.86
Renal Cancer	TK-10		-5.84
Breast Cancer	MDA-MB-231/ATCC	*	-5.81
Ovarian Cancer	OVCAR-8		-5.78
Non-Small Cell Lung Cancer	NCI-H226		-5.76
Renal Cancer	ACHN		-5.76
CNS Cancer	SF-539		-5.75
Leukemia	SR		-5.71
Colon Cancer	KM12		-5.70
Colon Cancer	SW-620	*	-5.68
Prostate Cancer	DU-145		-5.66
CNS Cancer	SF-268		-5.66
Colon Cancer	HCC-2998		-5.62
Renal Cancer	RXF 393		-5.61
Non-Small Cell Lung Cancer	HOP-62		-5.60
Renal Cancer	786-0		-5.59
Ovarian Cancer	SK-OV-3		-5.57
Colon Cancer	HT29		-5.46
Colon Cancer	COLO 205	*	-5.35
Ovarian Cancer	OVCAR-5	*	-5.26

Log10 High Conc :-4.0

National Cancer Institute Developmental Therapeutics Program<br>Waterfall Graph TGI	NSC : D - 765599/1	Units :Molar	SSPL :	EXP. ID :1207NS08
	Report Date :June 06, 2025		Test Date :July 16, 2012	

Panel	Cell Name	Hollow Fiber	TGI
Non-Small Cell Lung Cancer	HOP-92		-6.18
Leukemia	RPMI-8226		-6.02
Melanoma	UACC-62	*	-5.91
Melanoma	SK-MEL-5		-5.90
Melanoma	SK-MEL-2		-5.77
Renal Cancer	A498		-5.71
Non-Small Cell Lung Cancer	NCI-H23	*	-5.60
Leukemia	HL-60(TB)		-5.57
Colon Cancer	HCT-116		-5.55
Melanoma	LOX IMVI	*	-5.47
Leukemia	MOLT-4		-5.47
Melanoma	MALME-3M		-5.46
Renal Cancer	CAKI-1		-5.39
Breast Cancer	MDA-MB-468		-5.35
Melanoma	MDA-MB-435	*	-5.35
Renal Cancer	SN12C		-5.33
Breast Cancer	MDA-MB-231/ATCC	*	-5.32
Melanoma	UACC-257		-5.32
CNS Cancer	U251	*	-5.30
Melanoma	M14		-5.27
Prostate Cancer	PC-3		-5.27
Breast Cancer	BT-549		-5.26
Leukemia	CCRF-CEM		-5.25
Colon Cancer	HCC-2998		-5.23
Non-Small Cell Lung Cancer	NCI-H322M		-5.21
Renal Cancer	RXF 393		-5.17
Renal Cancer	TK-10		-5.16
Renal Cancer	UO-31		-5.16
Renal Cancer	786-0		-5.13
Non-Small Cell Lung Cancer	HOP-62		-5.13
Breast Cancer	T-47D		-5.13
Leukemia	SR		-5.08
Breast Cancer	HS 578T		-5.06
Melanoma	SK-MEL-28		-5.03
CNS Cancer	SNB-75		-5.01
Colon Cancer	HT29		-5.00
Ovarian Cancer	NCI/ADR-RES		-5.00
Colon Cancer	KM12		-5.00
Ovarian Cancer	OVCAR-8		-5.00
Non-Small Cell Lung Cancer	A549/ATCC		-4.97
Ovarian Cancer	IGROV1		-4.97
Non-Small Cell Lung Cancer	NCI-H460		-4.97
Colon Cancer	HCT-15		-4.94
Ovarian Cancer	SK-OV-3		-4.93
Non-Small Cell Lung Cancer	NCI-H226		-4.92
Breast Cancer	MCF7		-4.87
CNS Cancer	SF-539		-4.86
Colon Cancer	SW-620	*	-4.86
Renal Cancer	ACHN		-4.84
CNS Cancer	SNB-19		-4.82
Ovarian Cancer	OVCAR-3	*	-4.81
Colon Cancer	COLO 205	*	-4.81
Ovarian Cancer	OVCAR-4		-4.73
Leukemia	K-562		-4.72
Prostate Cancer	DU-145		-4.63
CNS Cancer	SF-268		-4.61
Ovarian Cancer	OVCAR-5	*	> -4.00

Log10 High Conc : -4.0

National Cancer Institute Developmental Therapeutics Program<br>Waterfall Graph LC50	NSC : D - 765599/1	Units :Molar	SSPL :	EXP. ID :1207NS08
	Report Date :June 06, 2025		Test Date :July 16, 2012	

Panel	Cell Name	Hollow Fiber	LC50
Melanoma	SK-MEL-5		-5.36
Melanoma	UACC-62	*	-5.14
Colon Cancer	HCT-116		-5.11
Melanoma	SK-MEL-2		-5.08
Melanoma	LOX IMVI	*	-4.68
Colon Cancer	HCC-2998		-4.67
CNS Cancer	U251	*	-4.53
Renal Cancer	UO-31		-4.49
Renal Cancer	RXF 393		-4.45
Colon Cancer	HT29		-4.44
Non-Small Cell Lung Cancer	HOP-62		-4.43
Renal Cancer	786-0		-4.40
Melanoma	MDA-MB-435	*	-4.37
Prostate Cancer	PC-3		-4.37
Colon Cancer	COLO 205	*	-4.35
Breast Cancer	BT-549		-4.31
Breast Cancer	MDA-MB-231/ATCC	*	-4.30
Non-Small Cell Lung Cancer	HOP-92		-4.27
CNS Cancer	SF-539		-4.20
Colon Cancer	KM12		-4.12
Renal Cancer	TK-10		-4.08
Melanoma	M14		-4.03
Non-Small Cell Lung Cancer	NCI-H23	*	-4.03
Non-Small Cell Lung Cancer	A549/ATCC		-4.00
Leukemia	CCRF-CEM		> -4.00
Leukemia	HL-60(TB)		> -4.00
Leukemia	K-562		> -4.00
Leukemia	MOLT-4		> -4.00
Leukemia	RPMI-8226		> -4.00
Leukemia	SR		> -4.00
Non-Small Cell Lung Cancer	NCI-H226		> -4.00
Non-Small Cell Lung Cancer	NCI-H322M		> -4.00
Non-Small Cell Lung Cancer	NCI-H460		> -4.00
Colon Cancer	HCT-15		> -4.00
Colon Cancer	SW-620	*	> -4.00
CNS Cancer	SF-268		> -4.00
CNS Cancer	SNB-19		> -4.00
CNS Cancer	SNB-75		> -4.00
Melanoma	MALME-3M		> -4.00
Melanoma	SK-MEL-28		> -4.00
Melanoma	UACC-257		> -4.00
Ovarian Cancer	IGROV1		> -4.00
Ovarian Cancer	OVCAR-3	*	> -4.00
Ovarian Cancer	OVCAR-4		> -4.00
Ovarian Cancer	OVCAR-5	*	> -4.00
Ovarian Cancer	OVCAR-8		> -4.00
Ovarian Cancer	NCI/ADR-RES		> -4.00
Ovarian Cancer	SK-OV-3		> -4.00
Renal Cancer	ACHN		> -4.00
Renal Cancer	CAKI-1		> -4.00
Renal Cancer	SN12C		> -4.00
Prostate Cancer	DU-145		> -4.00
Breast Cancer	MCF7		> -4.00
Breast Cancer	HS 578T		> -4.00
Breast Cancer	T-47D		> -4.00
Breast Cancer	MDA-MB-468		> -4.00

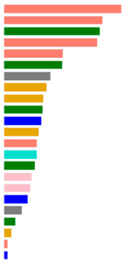

Log10 High Conc : -4.0
