# Supplementary material for: Integrated Pharmacogenomic and Structure-Guided Analyses Link LCC-10 (NSC765599) to an MMP-Associated Extracellular Matrix Regulatory Network in Leukemia
Source: Cells. 2026 Sep 4;15(17):1610. doi: 10.3390/cells15171610 (PMC13564721; doi:10.3390/cells15171610)
Supplement: Supplementary file 1 [file cells-15-01610-s001.zip › Supplementary Table S1_20260804_final.pdf]

Supplementary Table S1: Pharmacogenomic response similarity analysis of NSC765599 using DTP-COMPARE. Pearson's correlation coefficient (r) and common cell line count (CCLC) were used to quantify similarity between growth inhibition profiles across the NCI-60 panel. Synthetic compounds and clinically used anticancer agents are listed for comparison.

NCI synthetic compounds				
P	CCLC	Target NSC	Small molecule	MW (g/mol)
1	57	NSC765599	LCC-10	377.74
0.92	57	NSC765690	LCC-22	376.31
0.88	57	NSC765689	LCC-21	376.31
0.76	57	NSC765688	LCC-13	403.74
0.75	57	NSC765598	LCC-02	361.29
0.72	57	NSC50686	C.I. 37525	311.76
0.71	57	NSC758440	Niclosamide (USAN)	327.12
0.71	55	NSC753681	Derivative Benzothiazepine	175.25
0.7	54	NSC755806	XL147	541.02
0.68	57	NSC81947	Dibromsalan (USAN)	371.02
0.68	56	NSC781049	Isomammein	372.46
0.64	57	NSC754351	Exelixis PI3K inhibitor	270.29
0.61	56	NSC781046	Mammeisin	406.48
0.61	56	NSC760057	Nitazoxanide (USAN)	307.3
0.6	57	NSC757391	Dichlorophene	265.08
0.59	52	NSC406472	Octoxynol 9 (USAN)	602.68
0.59	52	NSC11227	Magneson II	293.28
NCI Standard agent				
P	CCLC	Target NSC	Marketed drug	Mechanism of action
0.29	57	NSC241240	CBDCA (Carboplatin)	Inhibiting DNA replication and transcription
0.25	57	NSC226080	Rapamycin	Inhibits mTOR pathway
0.21	56	NSC750	Busulfan	Cross-links DNA, preventing cell division
0.21	47	NSC249992	m-AMSA (Amsacrine)	Disrupts DNA replication and repair
0.21	57	NSC141540	VP-16 (Etoposide)	Prevents DNA repair and induces apoptosis.
0.2	56	NSC119875	Cisplatin	Alkylating agent

0.2	56	NSC63878	Cytosine arabinoside	Inhibits DNA polymerase, blocking DNA synthesis.
0.19	56	NSC122819	VM-26 (Teniposide)	Disrupts DNA replication
0.19	56	NSC82151	Daunorubicin (Daunomycin)	Inhibits topoisomerase II, causing DNA damage
0.18	56	NSC208734	Aclacinomycin A	Inhibits DNA and RNA synthesis
0.17	57	NSC123127	Doxorubicin (Adriamycin)	Intercalates DNA and inhibits topoisomerase II, inducing apoptosis.
0.17	56	NSC609699	Topotecan	Stabilizes DNA-topoisomerase complex, causing DNA breaks.
0.16	57	NSC3088	Chlorambucil	Alkylating agent
0.15	47	NSC95441	methyl-CCNU	Alkylating agent
0.14	56	NSC301739	DHAD (Mitoxantrone)	Inhibits topoisomerase II and intercalates DNA
0.14	56	NSC32065	Hydroxyurea	Inhibits ribonucleotide reductase
0.12	56	NSC264880	Dihydro-5-azacytidine	Inhibiting methylation and synthesis
0.12	56	NSC312887	Fludarabine phosphate	Inhibits DNA polymerase and RNA synthesis
