# Supplementary material for: Integrated Pharmacogenomic and Structure-Guided Analyses Link LCC-10 (NSC765599) to an MMP-Associated Extracellular Matrix Regulatory Network in Leukemia
Source: Cells. 2026 Sep 4;15(17):1610. doi: 10.3390/cells15171610 (PMC13564721; doi:10.3390/cells15171610)
Supplement: Supplementary file 1 [file cells-15-01610-s001.zip › Supplementary Table S2_20260804_final.pdf]

Supplementary Table S2: Predicted protein targets of LCC-10 (NSC765599) identified using SwissTargetPrediction. Predicted protein targets are grouped according to functional class, including proteases, receptor tyrosine kinases, serine/threonine kinases, enzymes, and secreted proteins. UniProt and ChEMBL identifiers are provided to facilitate target annotation and downstream structure-guided computational analyses.

Target	Name	Uniprot ID	ChEMBL ID	Functional class
**Matrix metalloproteinase 2**	**MMP2**	**P08253**	**CHEMBL333**	**Protease**
**Matrix metalloproteinase 3**	**MMP3**	**P08254**	**CHEMBL283**	**Protease**
**Matrix metalloproteinase 9**	**MMP9**	**P14780**	**CHEMBL321**	**Protease**
**Matrix metalloproteinase 12**	**MMP12**	**P39900**	**CHEMBL4393**	**Protease**
**Matrix metalloproteinase 13**	**MMP13**	**P45452**	**CHEMBL280**	**Protease**
Epidermal growth factor receptor erbB1	EGFR	P00533	CHEMBL203	Kinase
Fibroblast growth factor receptor 1	FGFR1	P11362	CHEMBL3650	Kinase
Platelet-derived growth factor receptor beta	PDGFRB	P09619	CHEMBL1913	Kinase
Vascular endothelial growth factor receptor 2	KDR	P35968	CHEMBL279	Kinase
MAP kinase p38 alpha	MAPK14	Q16539	CHEMBL260	Kinase
Serine/threonine-protein kinase mTOR	MTOR	P42345	CHEMBL2842	Kinase
Glycogen synthase kinase-3 beta	GSK3B	P49841	CHEMBL262	Kinase
Pyruvate dehydrogenase kinase isoform 1	PDK1	Q15118	CHEMBL4766	Kinase
Cyclin-dependent kinase 5/CDK5 activator 1	CDK5R1<br>CDK5	Q15078<br>Q00535	CHEMBL1907600	Kinase
Tyrosine-protein kinase SRC	SRC	P12931	CHEMBL267	Kinase
Protein tyrosine kinase 2 beta	PTK2B	Q14289	CHEMBL5469	Kinase
Serine/threonine-protein kinase Chk1	CHEK1	O14757	CHEMBL4630	Kinase
MAP kinase ERK2	MAPK1	P28482	CHEMBL4040	Kinase
Prostaglandin E synthase	PTGES	O14684	CHEMBL5658	Enzyme
Vascular endothelial growth factor A	VEGFA	P15692	CHEMBL1783	Secreted protein

Abbreviations: UniProt, Universal Protein Resource; ChEMBL, Chemical Database of Bioactive Molecules.
