# Supplementary material for: Integrated Pharmacogenomic and Structure-Guided Analyses Link LCC-10 (NSC765599) to an MMP-Associated Extracellular Matrix Regulatory Network in Leukemia
Source: Cells. 2026 Sep 4;15(17):1610. doi: 10.3390/cells15171610 (PMC13564721; doi:10.3390/cells15171610)
Supplement: Supplementary file 1 [file cells-15-01610-s001.zip › Supplementary Table S4_20260804_final.pdf]

Supplementary Table S4: Comprehensive in silico physicochemical, pharmacokinetic, medicinal chemistry, and toxicity-related (ADMET) prediction profiles of LCC-10 and representative matrix metalloproteinase inhibitors. Predictions were generated using SwissADME, ADMETLab 3.0, and pkCSM. Binary outputs (Yes/No) indicate model-based classifications, whereas numerical values represent predicted physicochemical parameters, probabilities, or risk scores.

**Drugs**	**LCC-10**	**Batimastat**	**Marimastat**	**PD 166793**	**SB-3CT**
Physicochemical properties					
Formula	$C_{21}H_{12}F_2N_2O_2$	$C_{23}H_{31}N_3O_4S_2$	$C_{15}H_{29}N_3O_5$	$C_{17}H_{18}BrNO_4S$	$C_{15}H_{14}O_3S_2$
MW (g/mol)	350.32	477.64	331.41	412.30	306.40
TPSA (Å)	73.12	161.07	127.76	91.85	77.05
Molar refractivity	92.57	127.95	84.86	96.47	81.14
Rotatable bonds	4	15	11	6	5
H-Bond acceptors	5	4	5	5	3
H-Bond donors	2	4	5	2	0
Lipophilicity and water solubility					
iLOGP	2.57	2.62	1.54	2.32	2.43
XLOGP3	5.47	3.55	0.51	4.16	3.18
MLOGP	3.72	2.05	-0.00	0.67	2.86
Consensus Log P	4.29	3.13	0.42	2.91	3.20
Log S (ESOL)	-5.71	-4.30	-1.49	-4.99	-3.86
Log S (SILICOS-IT)	-7.46	-6.14	-1.68	-5.90	-5.40
Pharmacokinetics					
GI absorption	High	Low	High	High	High
BBB permeant	No	No	No	No	No
P-gp substrate	No	Yes	No	Yes	No
CYP1A2 inhibitor	No	No	No	No	Yes

CYP2C9 inhibitor	Yes	Yes	No	Yes	Yes
CYP2D6 inhibitor	No	No	No	No	Yes
CYP3A4 inhibitor	No	Yes	No	No	Yes
log Kp	-4.55cm/s	-6.69	-7.96	-5.86	-5.91
Druglikeness					
Lipinski	Yes	Yes	Yes	Yes	Yes
Veber	Yes	No	No	Yes	Yes
Bioavailability score	0.55	0.55	0.55	0.56	0.55
Toxicity					
hERG blockers	0.843	0.225	0.018	0.114	0.212
DILI	0.98	0.987	0.422	0.991	0.998
AMES toxicity	0.854	0.875	0.967	0.052	0.792
Rat oral acute toxicity	0.766	0.739	0.304	0.193	0.166
FDAMDD	0.93	0.26	0.063	0.392	0.208
Skin sensitization	0.182	0.779	0.956	0.141	0.661
Carcinogenicity	0.298	0.087	0.671	0.368	0.717
Eye corrosion	0.0	0.0	0.0	0.003	0.004
Eye irritation	0.733	0.006	0.013	0.317	0.706
Respiratory	0.488	0.741	0.177	0.148	0.234
Human hepatotoxicity	0.96	0.881	0.621	0.575	0.838
