# Supplementary material for: Integrated Pharmacogenomic and Structure-Guided Analyses Link LCC-10 (NSC765599) to an MMP-Associated Extracellular Matrix Regulatory Network in Leukemia
Source: Cells. 2026 Sep 4;15(17):1610. doi: 10.3390/cells15171610 (PMC13564721; doi:10.3390/cells15171610)
Supplement: Supplementary file 1 [file cells-15-01610-s001.zip › Supplementary_Table_S3_restructured_S3A+B_final submit.pdf]

Supplementary Table S3. Docking setup, re-docking validation, AutoDock Vina docking scores, and representative ligand–protein interactions of LCC-10 (NSC765599) and reference MMP inhibitors across five representative MMP isoforms. Part A summarizes the receptor structures, docking-grid parameters, AutoDock Vina exhaustiveness setting, and re-docking RMSD values. Part B reports the predicted Vina docking scores and representative interactions identified from the top-ranked docking poses. Docking scores are computational predictions and should not be interpreted as experimental binding affinities, biochemical inhibition constants, or direct target engagement.

Part A. Receptor structures, docking-grid parameters, and re-docking validation

Receptor	PDB ID	Grid center (x, y, z)	Grid size (x × y × z, Å)	Exhaustiveness	Re-docking RMSD (Å)
MMP2	7XGJ	17.659, −15.958, 0.555	40 × 40 × 40	8	2.1506
MMP3	4JA1	1.820, 18.666, 20.127	40 × 40 × 40	8	0.8787
MMP9	6ESM	−0.434, 43.036, 14.868	40 × 40 × 40	8	1.9382
MMP12	3LJG	8.502, 15.032, 3.040	40 × 40 × 40	8	0.0353
MMP13	4JP4	26.403, 3.551, 16.072	40 × 40 × 40	8	2.2990

Notes: Grid-center coordinates and dimensions are reported in Å. The AutoDock Vina exhaustiveness parameter was set to 8. Re-docking RMSD values were calculated relative to the corresponding co-crystallized ligand poses. RMSD values <2.0 Å were obtained for MMP3, MMP9, and MMP12; MMP2 and MMP13 showed modestly higher deviations (2.1506 and 2.2990 Å, respectively). These validation results indicate receptor-dependent variability and are reported transparently rather than as evidence of quantitative binding accuracy.

Part B. Predicted docking scores and representative ligand–protein interactions

Compound	Receptor	PDB ID	Predicted Vina docking score (kcal/mol)	Representative predicted interactions (residues & distances)
NSC765599	MMP2	7XGJ	–10.1	Halogen: GLY A:136 (3.40 Å), PRO A:135 (3.27 Å); π–π T-shaped: TYR A:143 (5.0 Å), HIS A:121 (4.99 Å); π–alkyl: VAL A:118 (5.31 Å), ALA A:140 (5.06 Å)
NSC765599	MMP3	4JA1	–10.5	H- bonds: LEU A:218 (2.65 Å); Halogen: LEU A:226 (2.93Å), ASP A:228 (3.46Å), THR A:227 (3.25Å); π–alkyl: LEU A:218 (5.47Å); π–sigma: LEU A: 226 (3.63Å); π–π T-shaped: TYR A: 223 (5.45Å), HIS A:201 (4.41Å), PHE A:232 (4.77Å)
NSC765599	MMP9	6ESM	–11.1	H-bonds: TYR A:248 (2.31 Å), ARG A:249 (2.42 Å); C-H: ARG A:249 (2.42 Å); π–alkyl: LEU A: 222 (5.42Å), VAL A: 223 (5.16Å); π–anion: LEU A: 243 (3.77Å); π–cation: HIS A: 226 (4.69Å); π–sigma: LEU A: 243 (5.06Å), LEU A: 188 (4.82Å)
NSC765599	MMP12	3LJG	–11.5	H-bond: HIS A:218 (2.83Å), TYR A:240 (2.34Å); C-H: LEU A:181 (3.29Å); π–anion: HIS A:218 (4.29Å); π–π T-shaped: GLY A:179 (4.30Å); π–alkyl: ILE A:180 (5.48Å), LEU A:181 (5.07Å), LEU A:214 (5.02Å); π–sigma: VAL A:235 (3.82Å)
NSC765599	MMP13	4JP4	–9.9	H-bond: HIS A:232 (2.48Å), HIS A:222 (2.34Å), ALA A:186 (2.04Å); Halogen; ILE A:243 (3.05Å), PRO A:242 (3.65Å), PHE A:241 (2.77Å); π–π T-shaped: TYR A:244 (4.57Å), HIS A:222 (5.12Å), GLU A:223 (4.56Å); π–anion: GLU A:223 (4.15Å), HIS A:232 (4.97Å); π–alkyl: VAL A:219 (5.49Å, 4.34Å), LEU A:185 (5.07Å)
Batimastat	MMP2	7XGJ	–7.3	H-bonds: HIS A:121 (1.98Å), HIS A:131 (2.15Å), HIS A:125 (1.86Å), PRO A: 141 (2.17Å), LEU A:83 (2.15Å); π–σ: HIS A:125 (3.71Å); π–alkyl: VAL A:118 (5.11Å), LEU A:83 (4.93Å), HIS A:125 (4.29Å); C-H: GLY A:81 (3.54Å), LEU A:82 (3.15Å); π-sulfur: HIS A:131 (4.83Å); π-cation: HIS A:121 (3.94Å)
Batimastat	MMP3	4JA1	–8.2	H-bond: GLU A:216 (2.35Å), ARG A:233 (2.82Å), ASP A:238 (2.92Å), LEU A:218 (2.40Å); π–alkyl: LEU A:197 (4.31Å), LEU A:226 (4.72Å), HIS A:224 (4.84Å)
Batimastat	MMP9	6ESM	–8.2	H-bonds: PRO A:246 (2.11Å), HIS A:226 (2.67Å), ALA A:189 (2.44Å), GLN A:227 (3.02Å, 2.74Å); π–alkyl: LEU A:187 (4.86Å), LEU A:188 (5.36Å), PRO A:246 (4.61Å); π–sigma: TYR A:248 (3.84 Å)<br>C-H: ARG A:249 (3.19Å); π-sulfur: TYR A:248 (5.63Å), HIS A:226 (3.90Å)

Compound	Receptor	PDB ID	Predicted Vina docking score (kcal/mol)	Representative predicted interactions (residues & distances)
Batimastat	MMP12	3LJG	–9.1	H-bonds: PRO A:232 (2.23Å), LYS A:283 (2.79Å), ALA A:234 (2.48Å); C-H: LYS A:233 (3.60Å)<br>π–sigma: LYS A: (3.81Å); π–alkyll: LEU A: 214 (4.12Å), VAL A: 243 (3.43Å); π–cation & sulfur: HIS A:218 (3.69Å, 4.75Å); π–π T-shaped: TYR A:240 (5.37Å, 4.10Å)
Batimastat	MMP13	4JP4	–7.7	H-bonds: GLY A:183 (2.73Å), PRO A:242 (2.42Å), HIS A: 2.65 (2.65Å), HIS A: 222 (2.70Å); C-H: ALA A:186 (3.27Å), GLU A: 223 (3.56Å); π–alkyll: LEU A: 185 (5.08Å), VAL A: 219 (5.06Å); π–cation & anion: HIS A: 222 (4.84Å), HIS A:232 (4.96Å, 4.91Å); π–π T-shaped: TYR A: 244 (5.22Å)
Marimastat	MMP2	7XGJ	-6.9	H-bonds: ALA A:84 (2.41Å), HIS A:125 (2.73Å), HIS A:131 (2.52Å), HIS A:121 (2.41Å), ALA A:140 (1.77Å); π–alkyl: LEU A:82 (4.43Å), ALA A:122 (4.40Å), HIS A:125 (4.86Å), HIS A:131 (5.03Å), LEU A:83 (4.20Å)
Marimastat	MMP3	4JA1	-6.9	H-bonds: LEU A:218 (2.32Å), HIS A:224 (2.86Å), ARG A:233 (2.38Å); π–alkyl: LEU A:226 (4.67Å, 5.32Å), LEU A:197 (3.65Å)
Marimastat	MMP9	6ESM	-7.1	H-bonds: GLY A:186 (2.14Å), TYR A:248 (2.61Å), LEU A:188 (2.03Å), PRO A:246 (3.12Å, 2.96Å, 2.25Å), ALA A:189 (2.77Å), GLN A:227 (2.17Å); C-H: GLY A:186 (3.11Å); π–alkyl: TYR A:218 (4.89Å), LEU A:188 (4.45Å), HIS A:226 (3.62Å); π–sigma: HIS A:226 (4.15Å)
Marimastat	MMP12	3LJG	-7.8	H-bonds: ALA A:182 (2.32Å, 2.01Å), GLU A:219 (2.58Å), HIS A:222 (2.45Å), LEU A: 181 (2.13Å, 2.33Å), HIS A:228 (2.36Å), GLY A:179 (3.03Å), PRO A:238 (2.13Å); π–alkyl: ILE A:180 (4.90Å), LEU A:181 (5.25Å), HIS A:218 (3.90Å); π–sigma: HIS A:218 (3.73Å)
Marimastat	MMP13	4JP4	-6.9	H-bonds: ALA A:186 (2.80Å), GLU A:223 (1.86Å), LEU A:185 (2.46Å), HIS A:232 (1.93Å), HIS A:222 (2.03Å), HIS A:226 (1.98Å); C-H: LEU A:184 (3.55Å), HIS A:222 (3.27Å); π–alkyl: LEU A:184 (4.58Å), HIS A:222 (4.29Å), VAL A:219 (4.07Å); π–sigma: HIS A:222 (3.77Å)
PD 166793	MMP2	7XGJ	-9.6	H-bonds: GLU A:216 (2.06Å), ARG A:233 (2.17Å), SER A:235 (2.58Å); π–alkyl: LEU A:197 (5.02Å, 5.45Å), HIS A:201 (4.06Å), VAL A:198 (3.88Å); π–π T-shaped: HIS A:201 (4.93Å), TYR A:223 (5.12Å)
PD 166793	MMP3	4JA1	-9.2	H-bonds: HIS A:226 (2.77Å), GLN A:227 (2.22Å), ALA A:189 (2.32Å), HIS A:236 (2.44Å); C-H: ARG A:249 (3.05Å)
PD 166793	MMP9	6ESM	-10.9	π–alkyl: LEU A:222 (5.28Å, 5.00Å), ARG A:249 (5.30Å), LEU A:243 (4.59Å, 4.70Å), VAL A:223 (4.85Å), HIS A:230 (5.38Å), HIS A:236 (5.37Å); π–π T-shaped: HIS A:226 (5.29Å, 3.83Å)
PD 166793	MMP12	3LJG	-10.6	H-bonds: ALA A:182 (2.35Å), LEU A:181 (2.67Å); π–alkyl: VAL A:243 (5.12Å), PHE A:248 (4.31Å), LEU A:214 (4.86Å), VAL A:235 (5.46Å, 5.01Å); π–cation: HIS A:218 (4.23Å)
PD 166793	MMP13	4JP4	-9.7	H-bonds: GLU A:223 (2.81Å), ALA A:186 (2.81Å), HIS A:222 (2.50Å), HIS A:232 (2.31Å), PRO A: 242 (2.06Å); π–alkyl: HIS A:226 (5.23Å), HIS A:232 (4.86Å), VAL A:219 (4.53Å), LEU A:218 (4.79Å), LEU A:239 (5.42Å, 4.98Å); π–π T-shaped: TYR A:244 (5.33Å); π–cation: HIS A:222 (4.05Å)
SB-3CT	MMP2	7XGJ	-8.5	H-bonds: TYR A:143 (2.83Å); π–alkyl: LEU A:83 (5.11Å), VAL A:118 (4.45Å), HIS A:121 (5.04Å), ALA A:84 (4.24Å); π–π T-shaped: HIS A:121 (4.78Å), TYR A:143 (5.35Å), ALA A:137 (3.87Å)<br>π–sulfur: HIS A:121 (4.00Å)
SB-3CT	MMP3	4JA1	-8.2	H-bonds: HIS A:224 (2.19Å); π–alkyl: LEU A:218 (5.32Å), LEU A:164 (4.95Å); π–π T-shaped: TYR A:223 (5.11Å), HIS A: 201 (4.91Å, 4.75Å); π–sulfur: PHE A:232 (5.98Å)
SB-3CT	MMP9	6ESM	-9.3	H-bonds: HIS A:226 (2.66Å), GLN A:227 (2.70Å); π–alkyl: LEU A:187 (5.25Å), VAL A:223 (4.96Å), ARG A:249 (4.70Å), LEU A:222 (5.16Å); π–cation: HIS A:226 (4.26Å); π–sigma: LEU A:243 (3.90Å)
SB-3CT	MMP12	3LJG	-8.9	H-bonds: LYS A:241 (2.22Å); C-H: TYR A:240 (3.45Å); π–π T-shaped: HIS A:218 (4.05Å)
SB-3CT	MMP13	4JP4	-8.4	H-bonds: THR A:245 (3.00Å); π–alkyl: ALA A:186 (5.44Å), VAL A:219 (5.36Å, 4.84Å), LEU A: 218 (4.88Å); π–π T-shaped: TYR A:244 (4.33Å), HIS A:222 (5.18Å); π–sigma: LEU A:185 (3.95Å); π–cation: HIS A:222 (4.83Å); π–sulfur: PHE A:252 (5.23Å)

Abbreviations and interpretation: MMP, matrix metalloproteinase; RMSD, root mean square deviation; H-bond, conventional hydrogen bond; C–H, carbon–hydrogen bond. π-interaction labels are reported as identified in the docking-interaction analysis (e.g., π–alkyl, π–sigma, π–cation, π–anion, π–π T-shaped, and π–sulfur). Distances are in Å. The interaction entries in Part B are retained from the original Supplementary Table S3 and represent contacts predicted for the top-ranked AutoDock Vina poses. They are descriptive computational outputs and do not establish ligand residence, binding kinetics, direct target engagement, or enzymatic inhibition.
